# Adjunctive acupuncture for sepsis-associated acute gastrointestinal injury: a systematic review, meta-analysis, and exploratory Bayesian network meta-analysis

**DOI:** 10.3389/fmed.2026.1806453

**Published:** 2026-05-22

**Authors:** Xuemin Zhang, Hongyuan Sun, Yue Wen, Guiwei Li, Qingquan Liu

**Affiliations:** 1 Department of Infectious Diseases, First Teaching Hospital of Tianjin University of Traditional Chinese Medicine, Tianjin, China; 2 National Clinical Research Center for Chinese Medicine, Tianjin, China; 3 Beijing Hospital of Traditional Chinese Medicine, Capital Medical University, Beijing, China; 4 Beijing Institute of Chinese Medicine, Beijing, China

**Keywords:** acupuncture, acute gastrointestinal injury, gastrointestinal dysfunction, meta-analysis, sepsis

## Abstract

**Background:**

Acute gastrointestinal injury (AGI) is common in sepsis and is associated with multiple organ dysfunction and poor outcomes. Conventional supportive strategies often fail to restore gastrointestinal motility or adequately modulate systemic inflammation, underscoring the need for safe adjunctive interventions in critically ill patients.

**Methods:**

We conducted a systematic review and meta-analysis with an exploratory Bayesian network meta-analysis in accordance with the Preferred Reporting Items for Systematic Reviews and Meta-Analyses (PRISMA) guidelines. Randomized controlled trials and observational cohort studies enrolling adult patients with sepsis-associated gastrointestinal dysfunction were included. Acupuncture (electroacupuncture or manual acupuncture) as an adjunct to standard Western medical care was compared with standard care alone. No restrictions were applied to outcomes during the search phase. Risk of bias was assessed using the revised Cochrane Risk of Bias tool, version 2 (RoB 2) for randomized trials and the Risk of Bias in Non-randomized Studies of Interventions, Version 2 (ROBINS-I V2) for the non-randomized study. Pooled effect estimates were calculated using fixed- or random-effects models, with heterogeneity, publication bias, and sensitivity analyses (leave-one-out) assessed. An exploratory Bayesian network meta-analysis was also performed to compare electroacupuncture and manual acupuncture.

**Results:**

Twenty studies (19 randomized controlled trials and one retrospective cohort; *n* = 1,502) published between 2013 and 2025 were included. Adjunctive acupuncture was associated with improvements in gastrointestinal and physiological parameters, including reduced intra-abdominal pressure, lower Acute Physiology and Chronic Health Evaluation (APACHE) II scores, and increased bowel sounds. Procalcitonin was significantly reduced, while C-reactive protein and white blood cell count did not show statistically significant differences; heterogeneity was substantial for these inflammatory biomarkers. No statistically significant reduction in 28-day mortality was observed. The exploratory network meta-analysis did not detect convincing evidence of superiority between electroacupuncture and manual acupuncture. Reported adverse events were rare and mild.

**Conclusion:**

Current evidence suggests that acupuncture, when used as an adjunct to standard care, may improve gastrointestinal function and inflammatory profiles in patients with sepsis-associated AGI, but does not confer a clear survival benefit. Given methodological limitations and substantial heterogeneity, well-designed multicenter trials with rigorous controls and clinically relevant endpoints are warranted.

**Systematic review registration:**

https://www.crd.york.ac.uk/prospero/display_record.php?ID=CRD42024530297, Identifier CRD42024530297.

## Introduction

Sepsis remains a leading cause of mortality in intensive care units (ICUs) worldwide and continues to impose a substantial burden on healthcare systems (1–4). While circulatory and respiratory failures are traditionally prioritized, acute gastrointestinal injury (AGI) is increasingly identified as the “motor” of multiple organ dysfunction syndrome (MODS) (5–7). Impaired intestinal motility, feeding intolerance, and AGI occur in up to 60% of critically ill patients and are independently associated with secondary infection, prolonged ICU stay, and increased mortality (8, 9).

Despite the centrality of the gut in sepsis pathophysiology, therapeutic options remain limited. Current standard of care, including fluid resuscitation, early enteral nutrition, and pharmacological prokinetics (3, 10, 11), often fails to restore motility in severe sepsis. In addition, certain prokinetic agents may be associated with tachyphylaxis, and safety concerns vary among different drugs. For instance, domperidone carries a risk of QT interval prolongation, and metoclopramide is prone to induce extrapyramidal symptoms. This therapeutic deadlock highlights an urgent need for adjunctive strategies that can restore neuroenteric function without exacerbating hemodynamic instability.

Acupuncture has emerged as a potential non-pharmacological therapy for modulating gastrointestinal motility and systemic inflammation in critically ill patients (12). Experimental (13–16) and clinical (17–19) studies suggest that acupuncture may improve gastric emptying, enhance autonomic regulation, and activate the cholinergic anti-inflammatory pathway. However, the existing clinical evidence is fragmented, predominantly derived from small single-center trials with heterogeneous designs, acupuncture modalities, and outcome definitions. Importantly, the relative effectiveness of electroacupuncture versus manual acupuncture and their impact on clinically relevant outcomes, such as disease severity scores, inflammatory biomarkers, and mortality, remain unclear.

To address these knowledge gaps, we conducted a systematic review and meta-analysis, with an exploratory Bayesian network meta-analysis (NMA), to comprehensively evaluate the efficacy and safety of acupuncture as an adjunctive therapy for sepsis-associated gastrointestinal dysfunction in adult ICU patients. By synthesizing evidence across multiple clinical domains and comparing acupuncture modalities, this study aims to provide a more nuanced and clinically relevant assessment of acupuncture’s role in integrative sepsis management.

## Methods

### Study registration

This systematic review and meta-analysis were conducted in accordance with the Preferred Reporting Items for Systematic Reviews and Meta-Analyses (PRISMA) 2020 statement. The study protocol was prospectively registered in the International Prospective Register of Systematic Reviews (PROSPERO; registration number CRD42024530297).

## Eligibility criteria

### Inclusion criteria

Studies were eligible if they met the following criteria:

(1) Randomized controlled trials (RCTs) or observational cohort studies (prospective or retrospective);
(2) Adult patients aged ≥18 years;
(3) Confirmed diagnosis of sepsis based on established criteria [Sepsis-1.0 (20), Sepsis-2.0 (21), or Sepsis-3.0 (22)], accompanied by gastrointestinal dysfunction defined according to recognized consensus standards, including the 1992 American College of Chest Physicians (ACCP)/Society of Critical Care Medicine(SCCM) criteria (20) or the 2012 European Society of Intensive Care Medicine (ESICM) recommendations on AGI (23);
(4) All participants received standard Western medical care (3, 10, 11) consistent with contemporary guidelines (e.g., anti-infective therapy, fluid resuscitation, organ support, gastrointestinal mucosal protection, and symptomatic management), with the experimental group receiving acupuncture as an adjunctive intervention. No restrictions were imposed on acupoint selection, stimulation modality, treatment duration, or outcomes.

### Exclusion criteria

Studies were excluded if they met any of the following criteria:

(1) Inclusion of specific populations such as patients with malignancy, pregnancy, immunosuppressive therapy, or gastrointestinal dysfunction primarily attributable to trauma or intrinsic gastrointestinal disease;
(2) Unavailable full text or insufficient data for extraction;
(3) Duplicate publications or studies with overlapping datasets.

### Search strategy

A comprehensive literature search was conducted in PubMed, Embase, Web of Science, the Cochrane Library, China National Knowledge Infrastructure (CNKI), Wanfang Data, SinoMed, and the Chinese Scientific Journal Database (VIP) from database inception to November 11, 2025. The search strategy combined Medical Subject Headings (MeSH) terms and free-text keywords related to sepsis, gastrointestinal dysfunction, and acupuncture, without language restrictions. Reference lists of all included studies were manually screened to identify additional eligible publications. The detailed search strategies for all databases are provided in Supplementary File 3.

### Literature selection and data extraction

Two investigators (XZ and YW) independently screened titles, abstracts, and full texts for eligibility. Disagreements were resolved through discussion with a third investigator (HS). Data were independently extracted by two reviewers using a standardized form, including study characteristics, participant demographics, intervention details, outcome measures, and risk-of-bias domains. When necessary, corresponding authors were contacted to obtain missing information. Extracted data were cross-checked by additional investigators to ensure accuracy.

### Risk of bias assessment

The risk of bias in randomized controlled trials was assessed using the revised Cochrane Risk of Bias tool, version 2 (RoB 2) (24), while observational cohort studies were evaluated using the Risk of Bias in Non-randomized Studies of Interventions, Version 2 (ROBINS-I V2) (25). Any discrepancies were resolved by consensus or consultation with a third reviewer.

### Statistical analysis

Statistical analyses were performed using R software (version 4.5.2; R Foundation for Statistical Computing), with the *meta* (26), *BUGSnet* (27)*, arules,* and *arulesViz* packages. For pairwise meta-analyses, pooled effect estimates with 95% confidence intervals (CIs) were calculated using fixed- or random-effects models based on the magnitude of statistical heterogeneity. Dichotomous outcomes were summarized as odds ratios (ORs), and continuous outcomes as standardized mean differences (SMDs) or mean differences (MDs), as appropriate.

Statistical heterogeneity was assessed using the *χ*^2^ test and quantified with the *I*^2^ statistic, with values >50% indicating substantial heterogeneity. Publication bias was evaluated using funnel plots when at least ten studies were available. Sensitivity analyses were conducted using a leave-one-out approach. For studies reporting medians and interquartile ranges, means and standard deviations were estimated using the Quantile Estimation method (28).

An exploratory Bayesian NMA was performed to compare electroacupuncture and manual acupuncture. Fixed- and random-effects models were fitted, and model fit was evaluated using the deviance information criterion (DIC). Global inconsistency was assessed in networks with closed loops, and local inconsistency was examined using node-splitting models. Treatment rankings were estimated using the surface under the cumulative ranking curve (SUCRA) (29).

## Results

### Literature selection

**Figure 1:**
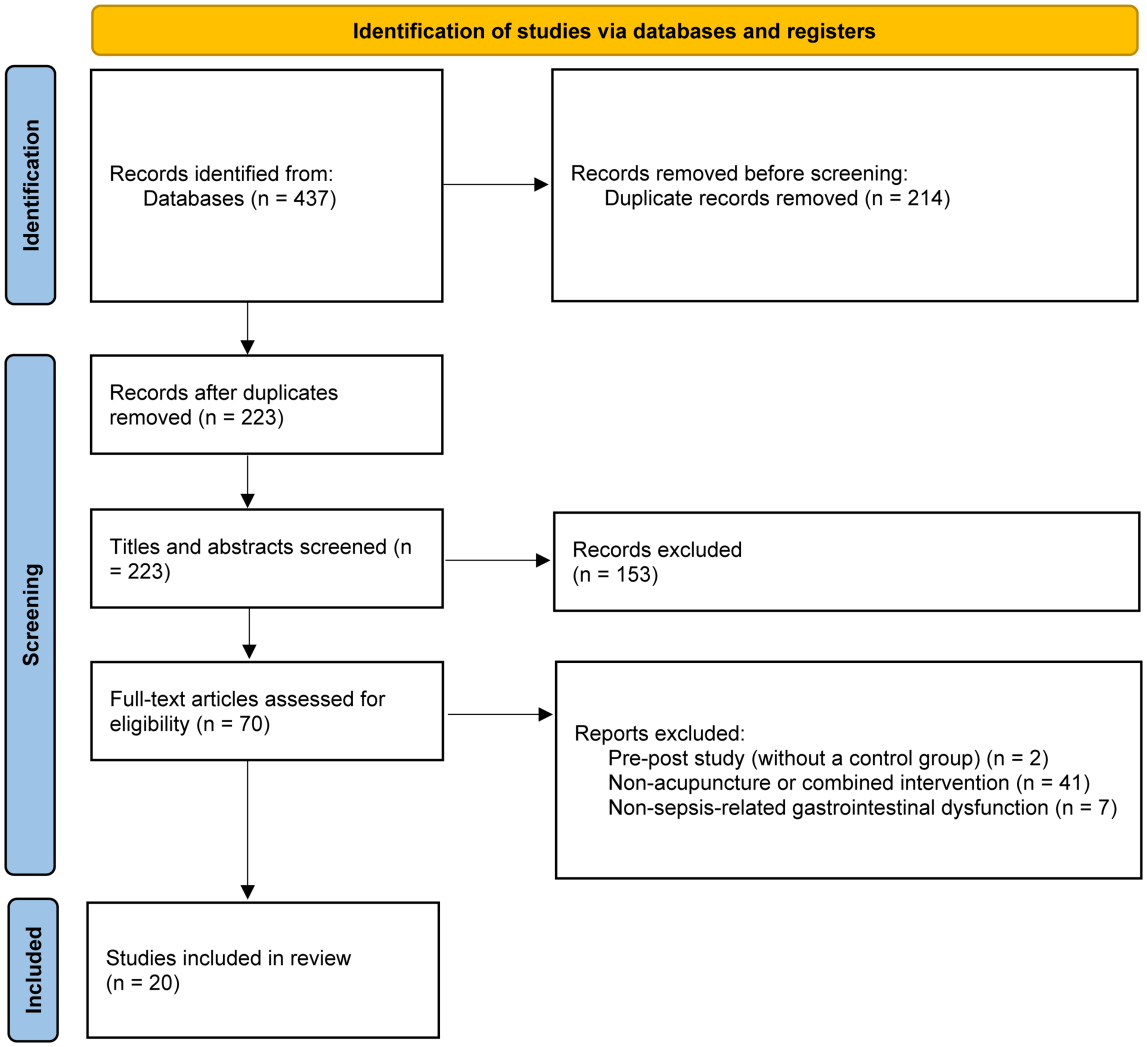
Flow diagram of study inclusion.

The literature search identified 437 records, of which 214 duplicates were removed. After screening titles and abstracts, 153 records were excluded. Full-text assessment was performed for 70 articles, and 50 were excluded for the following reasons: pre–post study design without a control group (*n* = 2), combined interventions (*n* = 41), or gastrointestinal dysfunction unrelated to sepsis (*n* = 7). Ultimately, 20 studies met the inclusion criteria (Figure 1), comprising 19 RCTs and 1 retrospective cohort study. The 19 RCTs were included in the pairwise meta‑analyses, and all 20 studies were included in the exploratory network meta‑analysis.

### Study characteristics

A total of 20 studies, including 19 randomized controlled trials and one retrospective cohort study, comprising 1,502 adult patients, were included. Sample sizes ranged from 30 to 150 participants. All studies were conducted in China between 2013 and 2025. Twelve studies evaluated electroacupuncture and eight evaluated manual acupuncture. Eighteen studies were published in Chinese and two in English. Although no language restrictions were applied during the search, only studies published in Chinese and English were identified. Literature in other languages was not retrieved and therefore not included in the analysis. Detailed study characteristics are summarized in Table 1.

**Table 1:** Characteristics of the studies included in meta-analysis.

Study	Country	Therapy		Acupoint	Acupuncture Protocol	Course (day)	Criteria	Outcomes
		T	C					
Yu et al. 2025 (30)	China	EA	WM	ST36, RN4	Continuous wave, maximum tolerable level, 30 min	7 day, bid	Sepsis 3.0/2012 ESICM	b, i
Sun et al. 2024 (35)	China	EA	WM	CV4, CV6, CV12, ST25, ST36, ST37, SP9	Deqi, dilatational wave, 2.7 mA, 20 min	14 day, qd	Sepsis 3.0/2012 ESICM	a, b, c, d, e, f, h
Dan et al. 2024 (32)	China	MA	WM	CV13, CV12, CV10, CV6, ST25, PC6, ST36	Deqi, retaining the needle, 30 min	5 day, qd	Sepsis 3.0/2012 ESICM	c, g, h
Xiaoju et al. 2022 (45)	China	MA	WM	CV10, CV12, CV13, ST25, GB34, ST36	Deqi, retaining the needle, 25 min	14 day, qd	Sepsis 3.0/2012 ESICM	c, d, e, i
Kai et al. 2022 (36)	China	EA	WM	CV12, CV10, CV4, CV6, ST36, ST26, ST24	Disperse-dense wave, 2 Hz, level that causes muscle fasciculation, 30 min	14 day, qd	Sepsis 3.0/2012 ESICM	a, g, h
Sun et al. 2021 (37)	China	EA	WM	ST36, ST37, ST25	Deqi, disperse-dense wave, maximum tolerable level, 30 min	7 day, bid	Sepsis 3.0/2012 ESICM	a, c, d, e, f, g, h
Lian et al. 2021 (49)	China	EA	WM	CV13, CV12, CV10, CV6, CV4, ST36, ST37, ST39	Disperse-dense wave, 2 Hz, maximum tolerable level, 30 min	7 day, qd	Sepsis 3.0/2012 ESICM	c, d, e
Yali et al. 2021 (46)	China	MA	WM	CV12, CV10, ST25, CV4, CV6, ST36, SP14	Deqi, retaining the needle, 20 min	7 day, qd	Sepsis 3.0/2012 ESICM	c, d, e, f, g
Liu et al. 2020 (38)	China	MA	WM	CV13, CV12, CV10, CV6, ST25, PC6, ST36	Deqi, retaining the needle, 20-30 min	7 day, qd	Sepsis 3.0/2012 ESICM	a, d, e, j
Sun et al. 2019 (44)	China	EA	WM	ST36, CV12, ST25, ST37, ST39	Deqi, continuous wave, 4 Hz, maximum tolerable level, 60 min	7 day, bid	Sepsis 3.0/2012 ESICM	b, c, d, e, g, h, i
Liu et al. 2019 (33)	China	EA	WM	CV12, ST25, SP15, ST24, ST26	Deqi, disperse-dense wave, maximum tolerable level, 1–1.5 Hz, 30 min	14 day, bid	Sepsis 2.0/2008 MODS (53)	a, d, e
Li et al. 2019 (39)	China	MA	WM	T6 - T12 EX-B2	Retaining the needle, 30 min	10 day, qd	Sepsis 2.0/1992 ACCP/SCCM	a, f, h
Wei et al. 2019 (47)	China	MA	WM	T6 - T12 EX-B2	Retaining the needle, 30 min	10 day, qd	Sepsis 2.0/1992 ACCP/SCCM	c, d, e
Meng et al. 2018 (31)	China	EA	WM	ST36, ST37	Deqi, continuous wave, the intensity was adjusted to induce visible muscle, 4 Hz, 20 min	5 day, bid	Sepsis 3.0/2012 ESICM	b, d, i, j
Jianfeng et al. 2017 (40)	China	EA	WM	ST36, ST37, ST25, CV12, PC6, CV6	Deqi, disperse-dense wave, 30 min	7 day, qd	Sepsis 3.0/2012 ESICM	a
Jun et al. 2016 (41)	China	MA	WM	ST36, ST37, CV12, CV6, PC6	Retaining the needle, 30 min	5 day, qd	Sepsis 2.0/2012 ESICM	a
Yihua et al. 2015 (42)	China	MA	WM	ST36, ST37, CV12, CV6, ST25, PC6	Retaining the needle, 30 min	5 day, qd	Sepsis 2.0/1992 ACCP/SCCM	a, c, d
Ruiying et al. 2015 (48)	China	EA	WM	ST36, CV4, ST25	Continuous wave, maximum tolerable level, 4 Hz, 30 min	7 day, bid	Sepsis 2.0/1992 ACCP/SCCM	c, d, e, f, g, h
Cai et al. 2014 (34)	China	EA	WM	ST36, ST25, ST37, ST39	Deqi, continuous wave, maximum tolerable level, 4 Hz, 60 min	5 day, bid	Sepsis 2.0/1992 ACCP/SCCM	b, c, e, g, i
Wu et al. 2013 (43)	China	EA	WM	ST36, ST25, ST37, ST39	Deqi, continuous wave, maximum tolerable level, 4 Hz, 60 min	3 day, bid	Sepsis 2.0/1992 ACCP/SCCM	a

a, the clinical effective rate; b, 28-day mortality; c, APACHE II score; d, intra-abdominal pressure; e, bowel sound; f, WBC; g, CRP; h, PCT; i, ICU length of stay; j, adverse events. EA, electroacupuncture; MA, manual acupuncture; WBC, white blood cell; CRP, C-reactive protein; PCT, procalcitonin; ICU, intensive care unit; WM, western medical; qd, once a day; bid, twice a day.

**Figure 2:**
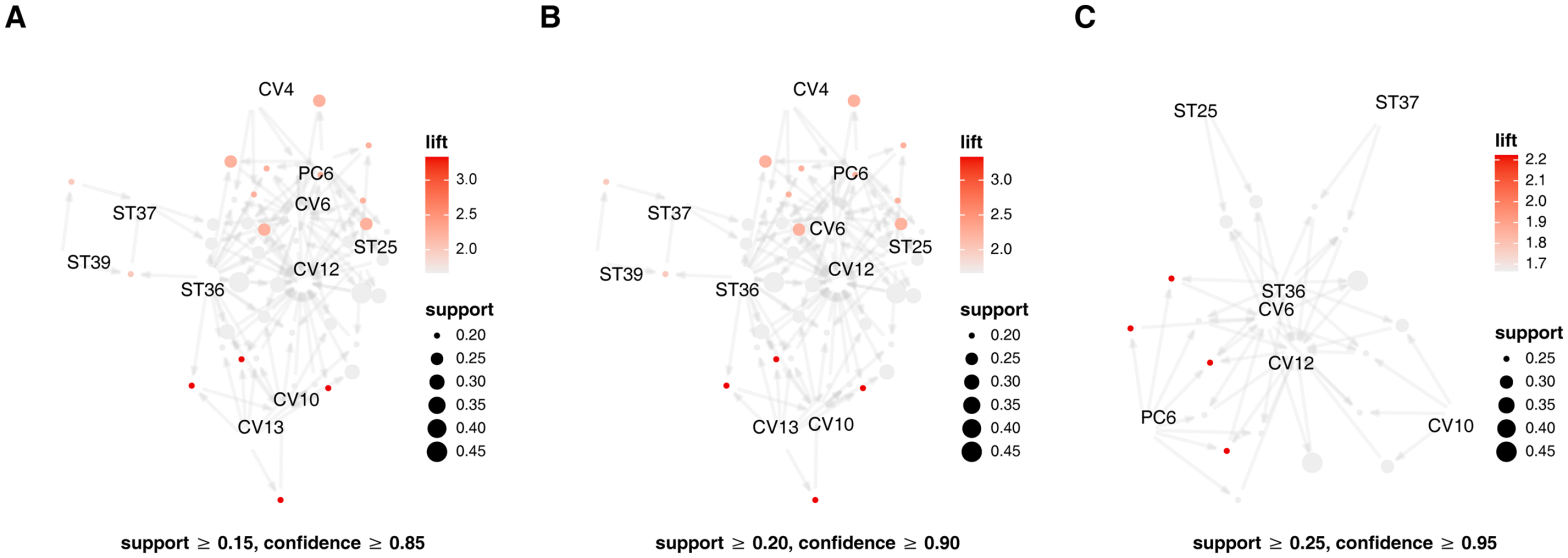
Association rules bubble chart and analysis chart of core prescription. **A–C** were the results of association analysis with different confidence levels and support levels, respectively.

Across the included studies, 18 distinct acupoints were reported. ST36, ST25, and CV12 were the most frequently used. Association rule analysis identified a core acupoint prescription consisting of ST36, ST25, CV12, ST37, CV6, CV10, and PC6 (Figure 2). The frequency of each acupoint was presented in Table 2. When the support threshold was ≥ 0.25 and the confidence level was ≥ 0.95, the results of the association rule analysis for acupoint prescriptions were shown in Table 3.

**Table 2:** Frequency acupoints for treating sepsis-associated acute gastrointestinal injury.

Number	Acupoint	Count	Frequency
1	ST36	17	17.71%
2	ST25	13	13.54%
3	CV12	12	12.50%
4	ST37	10	10.42%
5	CV6	9	9.38%
6	CV10	6	6.25%
7	CV4	5	5.21%
8	PC6	5	5.21%
9	CV13	4	4.17%
10	ST39	4	4.17%
11	EX-B2	2	2.08%
12	ST24	2	2.08%
13	ST26	2	2.08%
14	GB34	1	1.04%
15	RN4	1	1.04%
16	SP14	1	1.04%
17	SP15	1	1.04%
18	SP9	1	1.04%

**Table 3:** Analysis of association rules for acupoint prescription(support ≥ 0.25, confidence ≥ 0.95).

Number	Rules (lhs = > rhs)	Support	Confidence	Lift
1	{PC6} = > {CV6}	0.25	1.00	2.22
2	{CV12, PC6} = > {CV6}	0.25	1.00	2.22
3	{PC6, ST36} = > {CV6}	0.25	1.00	2.22
4	{CV12, PC6, ST36} = > {CV6}	0.25	1.00	2.22
5	{PC6} = > {CV12}	0.25	1.00	1.67
6	{CV10} = > {CV12}	0.30	1.00	1.67
7	{CV6} = > {CV12}	0.45	1.00	1.67
8	{CV6, PC6} = > {CV12}	0.25	1.00	1.67
9	{PC6, ST36} = > {CV12}	0.25	1.00	1.67
10	{CV10, CV6} = > {CV12}	0.25	1.00	1.67
11	{CV10, ST36} = > {CV12}	0.30	1.00	1.67
12	{CV6, ST37} = > {CV12}	0.25	1.00	1.67
13	{CV6, ST25} = > {CV12}	0.30	1.00	1.67
14	{CV6, ST36} = > {CV12}	0.45	1.00	1.67
15	{CV6, PC6, ST36} = > {CV12}	0.25	1.00	1.67
16	{CV10, CV6, ST36} = > {CV12}	0.25	1.00	1.67
17	{CV6, ST36, ST37} = > {CV12}	0.25	1.00	1.67
18	{CV6, ST25, ST36} = > {CV12}	0.30	1.00	1.67

### Risk of bias assessment

**Figure 3:**
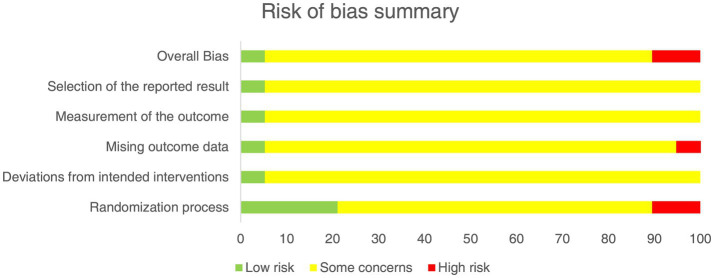
Risk of bias summary.

The retrospective cohort study (30) was judged to be at moderate risk of bias due to potential residual confounding, classification of interventions and selection of the reported result (Supplementary Table S1). Among the 19 RCTs, one (31) was assessed as low risk of bias, three (32–34) as high risk, and the remainder as having some concerns, primarily related to randomization procedures and incomplete outcome data (Figure 3; Supplementary Figure S1).

### Outcomes

#### The clinical effective rate

**Figure 4:**
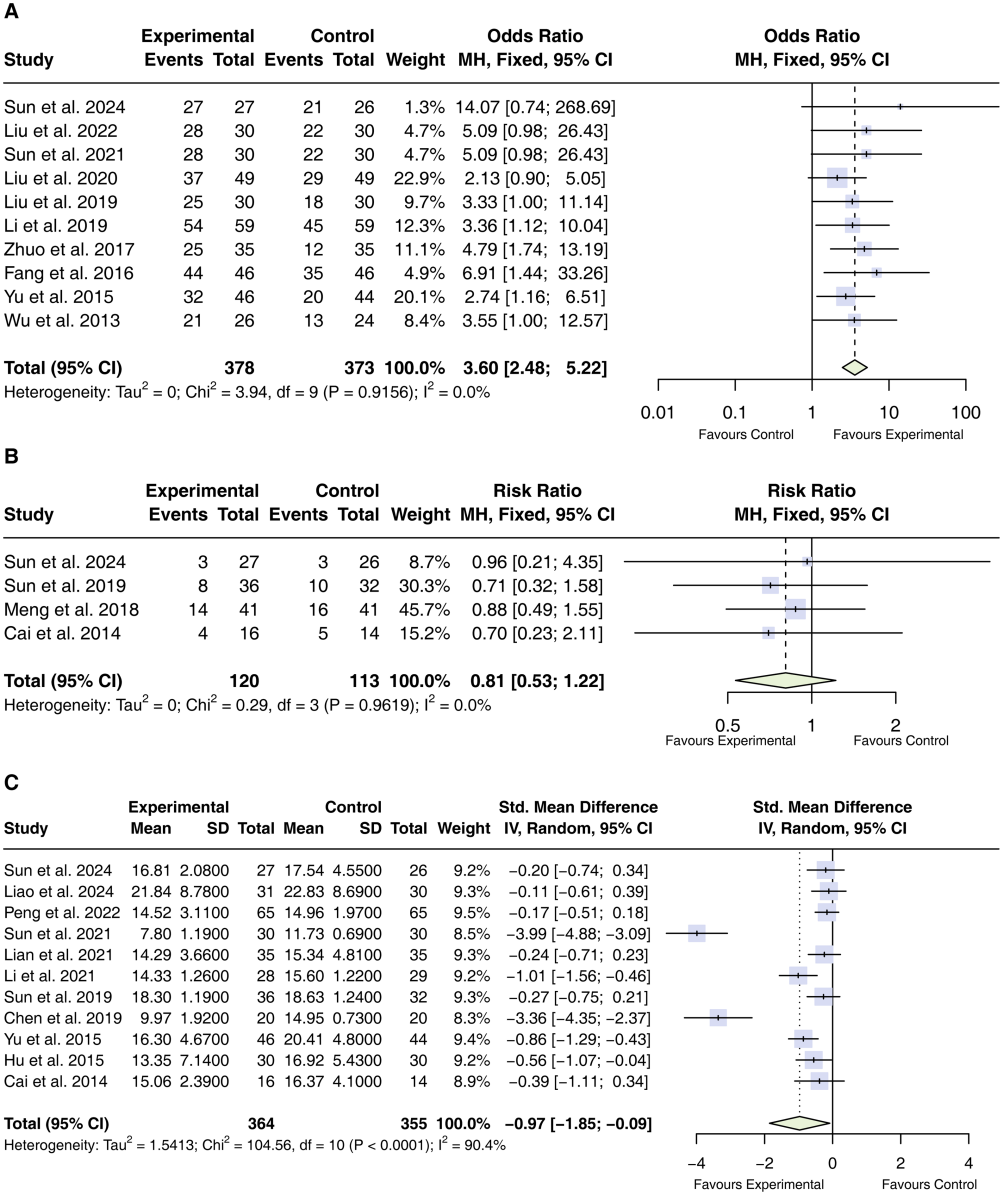
Forest plot of the clinical effective rate, 28-day mortality, and APACHE II score. **(A)** The clinical effective rate; **(B)** 28-day mortality; **(C)** APCHE II score. CIs, confidence intervals.

Ten RCTs (33, 35–43) involving 751 participants reported clinical effectiveness. No significant heterogeneity was observed (*I^2^* = 0%). Acupuncture as an adjunctive therapy was associated with a higher clinical effective rate compared with WM alone (OR = 3.60, 95% CI 2.48 to 5.22; Figure 4A).

#### 28-day mortality

Four RCTs (31, 34, 35, 44) (233 participants) reported 28-day mortality. Heterogeneity was negligible (*I^2^* = 0%). The pooled analysis did not demonstrate a statistically significant difference between groups (RR = 0.81, 95% CI: 0.53 to 1.22; Figure 4B).

#### Acute physiology and chronic health evaluation (APACHE) II score

Eleven studies (32, 34, 35, 37, 42, 44–48) (719 participants) reported APACHE II scores. Substantial heterogeneity was observed (*I^2^* = 90.4%); therefore, a random-effects model was applied. Adjunctive acupuncture was associated with lower APACHE II scores compared with standard care alone (SMD = −0.97, 95% CI: −1.85 to −0.09; Figure 4C).

#### Intra-abdominal pressure (IAP)

**Figure 5:**
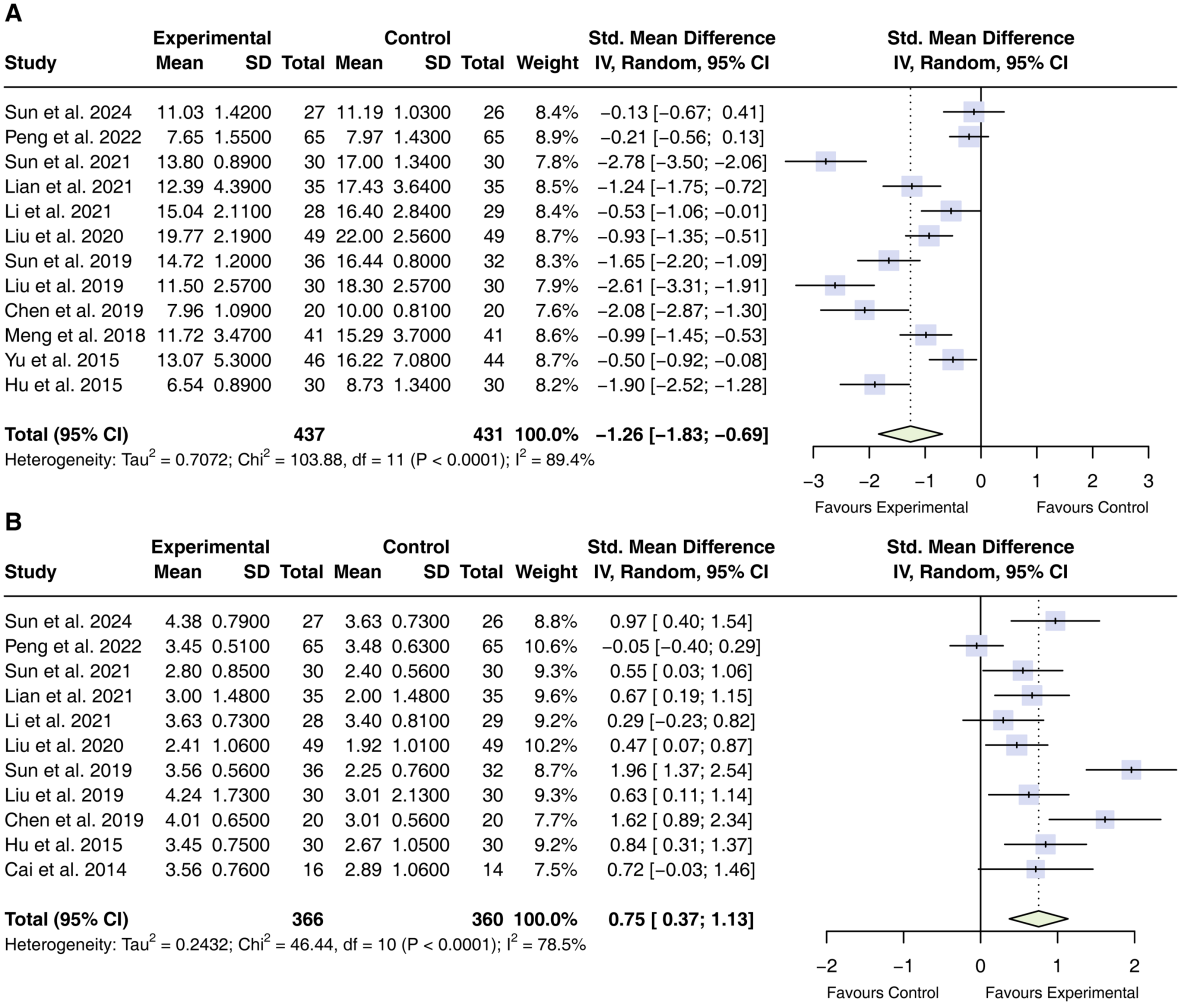
Forest plot of intra-abdominal pressure and bowel sound. **(A)** Intra-abdominal pressure; **(B)** bowel sound. CIs, confidence intervals.

Twelve studies (31, 33, 35, 37, 38, 42, 44–49) involving 868 participants reported data on IAP. Substantial heterogeneity was observed among the included trials (*I*^2^ = 89.4%); therefore, a random-effects model was applied. The pooled analysis showed that adjunctive acupuncture was associated with lower IAP compared with standard care alone (SMD = −1.26, 95% CI −1.83 to −0.69; Figure 5A).

#### Bowel sound

Eleven studies (33–35, 37, 38, 44–49) comprising 726 participants reported bowel sound outcomes. Given the substantial heterogeneity across studies (*I*^2^ = 78.5%), a random-effects model was used. Adjunctive acupuncture was associated with increased bowel sounds compared with standard care alone (SMD = 0.75, 95% CI 0.37 to 1.13; Figure 5B).

#### White blood cell (WBC) count

**Figure 6:**
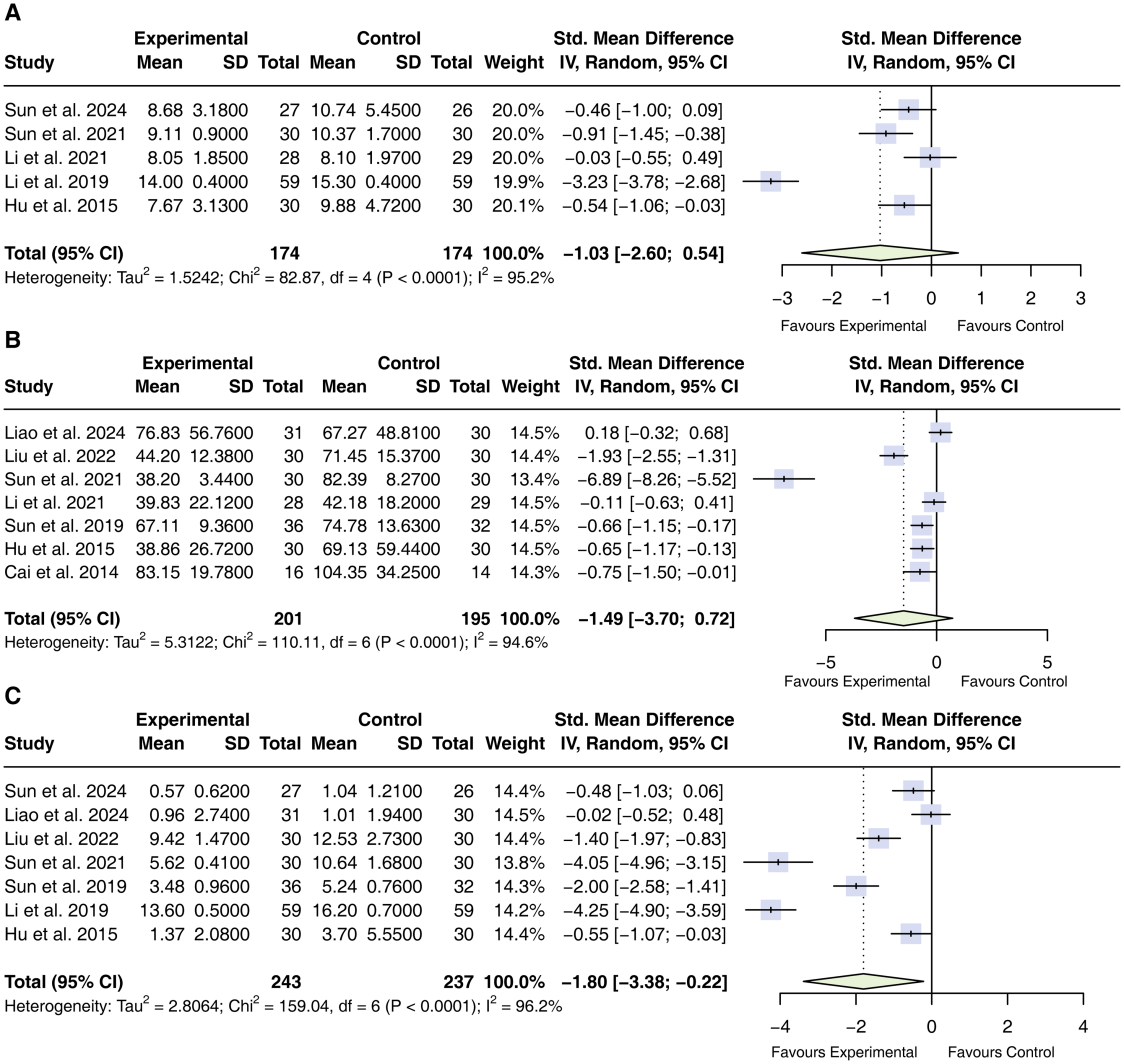
Forest plot of WBC, CRP, and PCT. **(A)** WBC; **(B)** CRP; **(C)** PCT. WBC, white blood cell; CRP, C-reactive protein; PCT, procalcitonin; CIs, confidence intervals.

Five studies (35, 37, 39, 46, 48) involving 348 participants reported WBC counts. Considerable heterogeneity was present (*I*^2^ = 95.2%); accordingly, a random-effects model was applied. The pooled analysis did not demonstrate a statistically significant difference in WBC counts between adjunctive acupuncture and standard care alone (SMD = −1.03, 95% CI −2.60 to 0.54; Figure 6A).

#### C-reactive protein (CRP)

Seven studies (32, 34, 36, 37, 44, 46, 48) comprising 396 participants reported CRP levels. Substantial heterogeneity was observed (*I*^2^ = 94.6%); therefore, a random-effects model was employed. The pooled analysis did not demonstrate a statistically significant difference in CRP levels between adjunctive acupuncture and standard care alone (SMD = −1.49, 95% CI -3.70 to 0.72; Figure 6B).

#### Procalcitonin (PCT)

Seven studies (32, 35–37, 39, 44, 48) including 480 participants reported procalcitonin levels. Owing to substantial heterogeneity among trials (*I*^2^ = 96.2%), a random-effects model was used. The pooled analysis showed that adjunctive acupuncture was associated with lower PCT levels compared with standard care alone (SMD = −1.80, 95% CI −3.38 to −0.22; Figure 6C).

#### ICU length of stay

**Figure 7:**
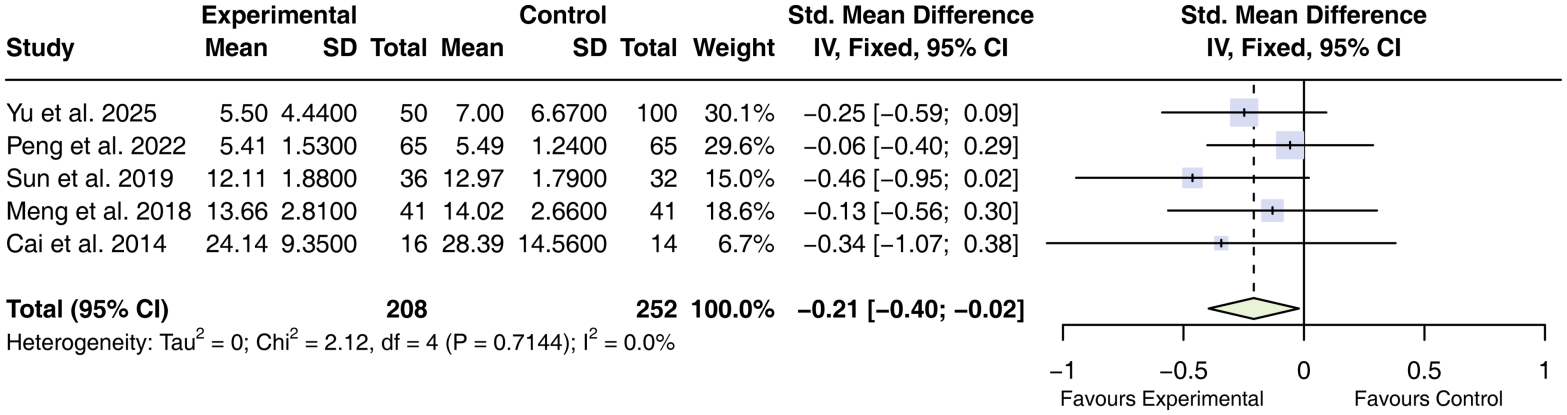
Forest plot of ICU length of stay. Note: ICU, intensive care unit; CIs, confidence intervals.

Five studies (30, 31, 34, 44, 45) reported ICU length of stay, with pooled results indicating a modest reduction associated with acupuncture (SMD = −0.21, 95% CI −0.40 to −0.02; *I*^2^ = 0%; Figure 7).

#### ADRs/ADEs

Information on adverse events was reported in only two of the included studies. One study (31) reported no adverse events in either group. The other study (38) reported two cases of transient somnolence in the acupuncture group and two cases of diarrhea and one case of somnolence in the control group; all events resolved spontaneously without specific intervention. The remaining 18 studies did not provide explicit information regarding adverse events.

#### Funnel plot characteristics

**Figure 8:**
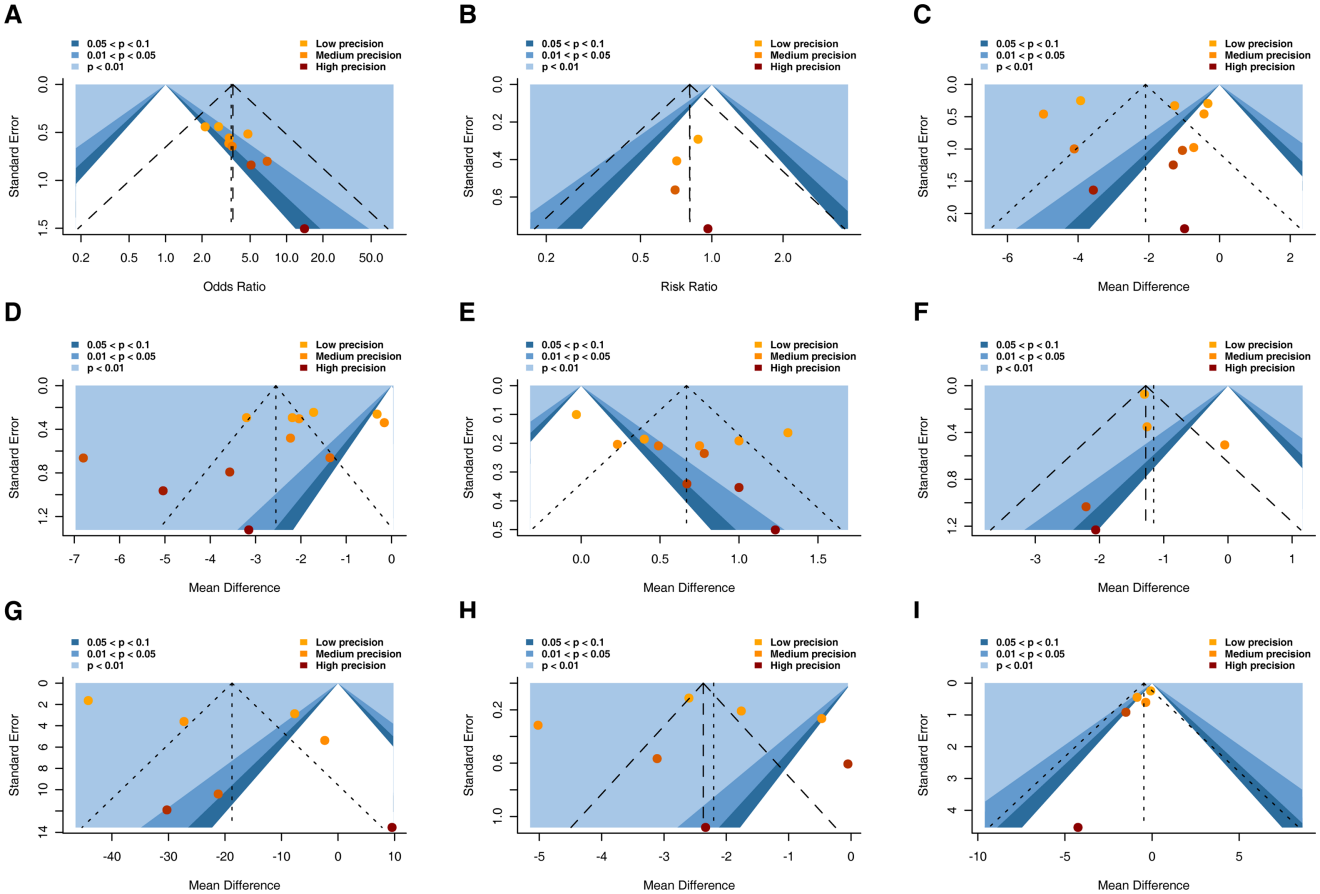
The funnel plot for different outcomes. **(A)** The clinical effective rate; **(B)** 28-day mortality; **(C)** APACHE II score; **(D)** intra-abdominal pressure; **(E)** bowel sound; **(F)** WBC; **(G)** CRP; **(H)** PCT; **(I)** ICU length of stay. WBC, white blood cell; CRP, C-reactive protein; PCT, procalcitonin; ICU, intensive care unit.

Contour-enhanced funnel plots were generated for each outcome (Figure 8). Visual inspection suggested relative symmetry for the clinical effective rate, 28-day mortality, APACHE II score, Bowel sound, WBC, and ICU length of stay. In contrast, funnel plots for IAP, CRP, and PCT showed a paucity of studies in regions corresponding to non‑significant effects, indicating a potential risk of publication bias.

#### Certainty of the evidence

The Grades of Recommendation, Assessment, Development, and Evaluation (GRADE) approach was employed to assess the certainty of the evidence for all outcomes. The certainty of evidence regarding 28-day mortality was graded as very low, whereas the evidence for all remaining outcomes was graded as low. Detailed GRADE evidence profiles are summarized in Supplementary Table S2.

#### Sensitivity analysis

Leave-one-out sensitivity analyses showed that the pooled effect size for clinical efficacy was robust, with consistent effect directions following the sequential exclusion of individual studies (Supplementary Figure S2A). Nevertheless, interpretation of this outcome warrants caution because clinical efficacy represents a composite and non-standardized measure. For 28-day mortality, sensitivity analyses excluding the observational study yielded consistent effect estimates (Supplementary Figure S2B), indicating stable results. In contrast, the pooled effect for the APACHE II score was sensitive to the exclusion of three specific studies; removal of any one of these studies resulted in loss of statistical significance, suggesting limited robustness of this outcome (Supplementary Figure S2C). Regarding physiological outcomes, the pooled effects for intra-abdominal pressure and bowel sounds remained robust, with unchanged effect directions after sequential removal of individual studies (Supplementary Figure S3). Similarly, the pooled estimates for WBC, CRP, PCT, and ICU length of stay demonstrated limited robustness (Supplementary Figures S4, S5).

#### Exploratory network meta-analysis

**Figure 9:**
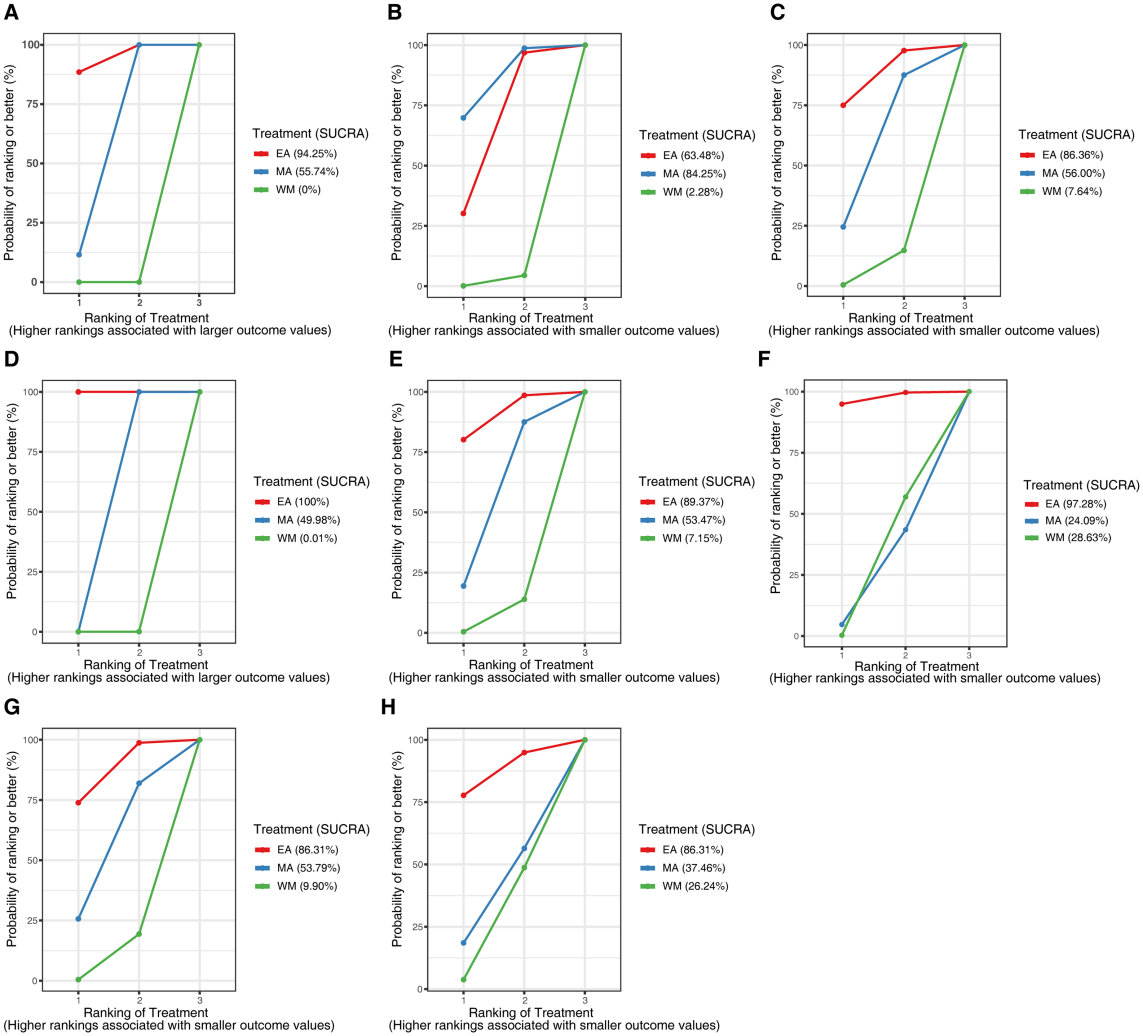
Plot of SUCRA for different outcomes. **(A)** The clinical effective rate; **(B)** APACHE II score; **(C)** intra-abdominal pressure; **(D)** bowel sound; **(E)** WBC; **(F)** CRP; **(G)** PCT; **(H)** ICU length of stay. SUCRA, surface under the cumulative ranking curve; WBC, white blood cell; CRP, C-reactive protein; PCT, procalcitonin; ICU, intensive care unit.

**Figure 10:**
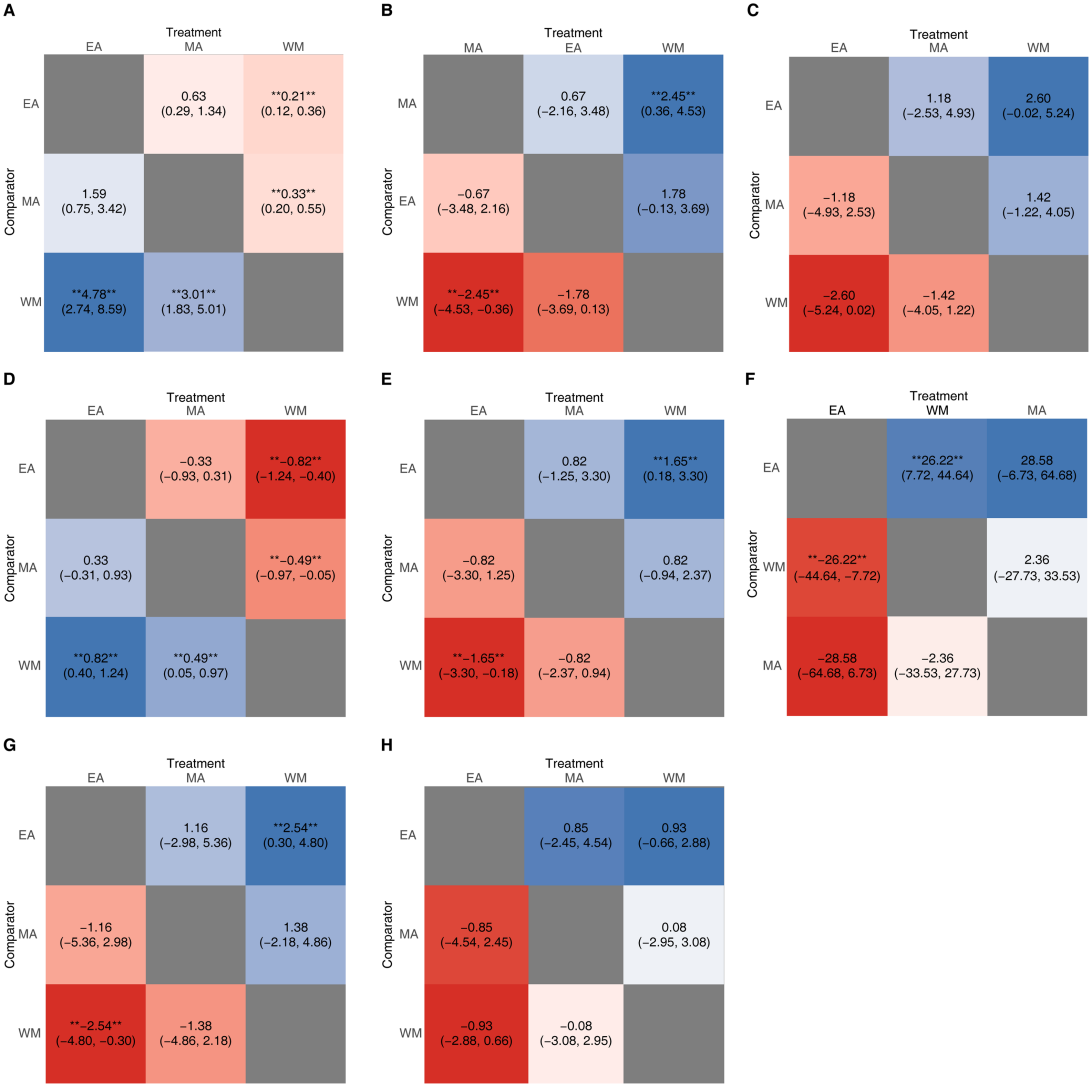
The league heat plot for different outcomes. **(A)** the clinical effective rate; **(B)** APACHE II score; **(C)** intra-abdominal pressure; **(D)** bowel sound; **(E)** WBC; **(F)** CRP; **(G)** PCT. **(H)** ICU length of stay. Results are presented as OR with95% CIs or MD with 95% CIs. The values in each cell represent the relative effect of the treatment on the top, compared to the treatment on the left. A double asterisk indicates statistical significance. SUCRA, surface under the cumulative ranking curve; OR, odds ratios; MD, mean differences; CIs, confidence intervals; WBC, white blood cell; CRP, C-reactive protein; PCT, procalcitonin; ICU, intensive care unit.

To investigate the comparative efficacy of electroacupuncture (EA) and manual acupuncture (MA), an exploratory NMA was conducted for all outcome measures, excluding 28-day mortality. The network plots illustrating the evidence structure for these outcomes are presented in Supplementary Figure S6. Comprehensive analysis of the SUCRA values (Figure 9) and league table heat maps (Figure 10) revealed no convincing evidence of superiority between EA and MA across any of the evaluated endpoints.

## Discussion

In this comprehensive synthesis of 1,502 patients, we evaluated acupuncture as an adjunctive intervention for sepsis-associated acute gastrointestinal injury. The findings indicate that adjunctive acupuncture was associated with improvements in gastrointestinal function and systemic inflammatory profiles, including reductions in IAP, APACHE II scores, and procalcitonin levels. However, reductions in C-reactive protein and white blood cell count did not reach statistical significance, and these physiological and biochemical benefits did not translate into a statistically significant reduction in 28-day mortality.

The observed dissociation between physiological improvement and survival benefit underscores a phenomenon of physiological-outcome decoupling in advanced sepsis. While acupuncture may modulate autonomic balance and attenuate gastrointestinal-derived inflammatory signaling, such effects are unlikely to reverse established multi-organ failure once irreversible microcirculatory dysfunction or cellular metabolic failure has occurred. Moreover, mortality in sepsis represents a competing and multifactorial endpoint, influenced by pathogen characteristics, timeliness of antimicrobial therapy, and host responses, which may dilute the detectable impact of adjunctive interventions. Consequently, even this large pooled analysis may be insufficiently powered to identify modest effects on short-term survival.

Substantial statistical heterogeneity was observed, particularly for inflammatory biomarkers (*I*^2^ > 90%), although the direction of effects was generally consistent across studies. Potential contributors to heterogeneity include variations in sepsis definitions (Sepsis-1/2 versus Sepsis-3), baseline disease severity, timing and duration of acupuncture intervention, and differences in concurrent ICU management. In addition, the clinical effective rate, frequently reported in Chinese trials, represents a composite and non-standardized outcome and should therefore be interpreted with caution.

The exploratory Bayesian network meta-analysis did not detect statistically robust differences between electroacupuncture and manual acupuncture. Although this finding challenges the assumption that electrical stimulation is inherently required for therapeutic benefit, the network structure was sparse and lacked closed loops, precluding the assessment of inconsistency. Therefore, these results should be interpreted with caution, and no definitive conclusion can be drawn regarding the relative importance of acupoint selection versus stimulation modality. These findings require validation in future head-to-head trials.

This systematic review simultaneously pools data on both gastrointestinal functional outcomes and systemic inflammatory biomarkers in sepsis-associated AGI and formally compares electroacupuncture and manual acupuncture through an exploratory network meta-analysis. Previous meta-analyses on acupuncture for sepsis-related gastrointestinal dysfunction have either focused on a single acupuncture modality or aggregated a narrower set of endpoints without examining comparative efficacy. By including a broader range of clinically relevant outcomes and employing network meta-analytic techniques, this study provides a more nuanced evidence base to inform the design of future integrative critical care trials.

These clinical benefits align with the neuroanatomical basis of the cholinergic anti-inflammatory pathway (50). Stimulation of ST36 recruits somatic afferents that project to the dorsal motor nucleus of the vagus nerve. This efferent signaling activates *α*-nicotinic acetylcholine receptors (α7nAChR) on splenic macrophages, thereby inhibiting the transcription of NF-κB and subsequent release of TNF-α and IL-6 (51, 52). This “hard-wired” reflex provides a biological plausibility for the observed reduction in inflammatory biomarkers and intra-abdominal pressure, effectively interrupting the vicious cycle of barrier dysfunction and bacterial translocation.

An important limitation of acupuncture in critically ill patients is the difficulty in confirming the “Deqi” response in sedated or comatose individuals. In such patients, subjective feedback on needle sensation is unavailable, resulting in potential variability in the intensity and duration of needling, as well as in individual sensitivity to stimulation. This uncertainty may lead to discrepancies between the intended therapeutic effect and the actual physiological response, thereby potentially affecting both physiological parameters and clinical outcomes. Moreover, this factor may act as a confounding variable, introducing uncertainty into evaluations of acupuncture efficacy. Therefore, when assessing the value of acupuncture in ICU, the limitations associated with patients’ levels of consciousness should be carefully considered. Future studies should explore quantifiable or alternative approaches for assessing stimulation intensity, such as neurophysiological markers or automated monitoring of needle stimulation, to improve the accuracy and reproducibility of efficacy evaluations.

Several limitations merit consideration. Most included studies were conducted in China, which may limit generalizability to other healthcare systems. The overall methodological quality of the evidence was moderate, with few trials at low risk of bias. Reporting of adverse events was insufficient, precluding firm conclusions regarding safety in critically ill patients. Additionally, funnel plot asymmetry for inflammatory outcomes suggests potential publication bias, which may have led to overestimation of treatment effects.

## Conclusion

In summary, current evidence supports acupuncture as a biologically plausible, effective adjunct for mitigating gastrointestinal dysfunction and dampening systemic inflammation in sepsis. While it does not currently stand as a mortality-reducing intervention, its potential to improve organ-specific metrics warrants its consideration in integrative sepsis management protocols. Future efforts should prioritize multicenter, sham-controlled trials powered specifically for hard clinical endpoints, utilizing biomarker-guided stratification to identify the “hyper-inflammatory” phenotypes most likely to benefit from neuro-modulation.

## Data Availability

The original contributions presented in the study are included in the article/Supplementary material, further inquiries can be directed to the corresponding author/s.

## Author’s note

Guiwei Li and Qingquan Liu jointly supervised this work.
