# Supplementary material for: Adjunctive acupuncture for sepsis-associated acute gastrointestinal injury: a systematic review, meta-analysis, and exploratory Bayesian network meta-analysis
Source: Front Med (Lausanne). 2026 May 22;13:1806453. doi: 10.3389/fmed.2026.1806453 (PMC13237696; doi:10.3389/fmed.2026.1806453)
Supplement: Supplementary file 1 [file Data_Sheet_1.docx]

[**Table S1. The risk of bias assessment for retrospective cohort study.** 2](#_Toc228224330)

[**Table S2. GRADE Summary of Findings.** 3](#_Toc228224331)

[**Figure S1. Risk of bias graph for Randomized controlled trials.** 4](#_Toc228224332)

[**Figure S2. The results of sensitivity analysis for the clinical effective rate, 28-day mortality and APCHE II score.** 5](#_Toc228224333)

[**Figure S3. The results of sensitivity analysis for intra-abdominal pressure and bowel sound.** 6](#_Toc228224334)

[**Figure S4. The results of sensitivity analysis for WBC, CRP and PCT.** 7](#_Toc228224335)

[**Figure S5. The results of sensitivity analysis for ICU length of stay.** 7](#_Toc228224336)

[**Figure S6. The Network plot of the different outcomes.** 8](#_Toc228224337)

[**Figure S7. The Assess model fit of the different outcomes.** 9](#_Toc228224338)

[**Table S3. Details of the control group interventions** 10](#_Toc228224339)

**Table S1. The risk of bias assessment for retrospective cohort study.**

| Domain | Risk level |
| --- | --- |
| D1: Bias due to confounding | Medium |
| D2: Bias in classification of interventions | Medium |
| D3: Bias in selection of participants | Low |
| D4: Bias due to missing data | Low |
| D5: Bias in measurement of the outcome | Low |
| D6: Bias in selection of the reported result | Medium |

**Table S2. GRADE Summary of Findings.**

| Outcomes | No. of Studies | Participants | Certainty of Evidence | |
| --- | --- | --- | --- | --- |
| Clinical effective rate | 10 | 751 | ⨁⨁◯◯ | Low |
| 28-day Mortality | 5 | 383 | ⨁◯◯◯ | Very Low |
| APACHE II | 11 | 719 | ⨁⨁◯◯ | Low |
| Intra-abdominal Pressure | 12 | 868 | ⨁⨁◯◯ | Low |
| Bowel Sound | 11 | 726 | ⨁⨁◯◯ | Low |
| WBC | 5 | 348 | ⨁⨁◯◯ | Low |
| CRP | 7 | 396 | ⨁⨁◯◯ | Low |
| PCT | 7 | 480 | ⨁⨁◯◯ | Low |
| ICU Length of Stay | 5 | 460 | ⨁⨁◯◯ | Low |


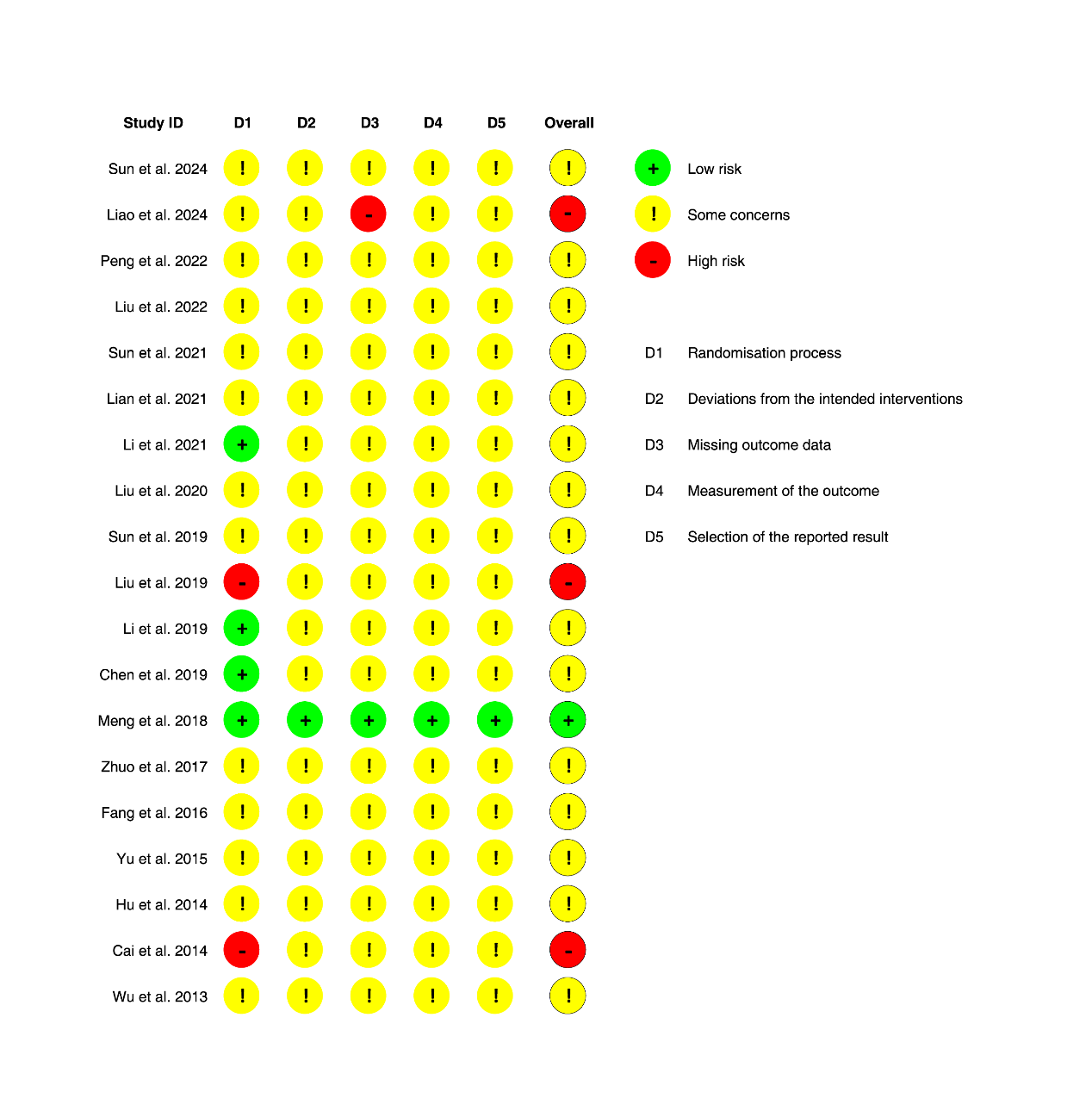


**Figure S1. Risk of bias graph for Randomized controlled trials.**


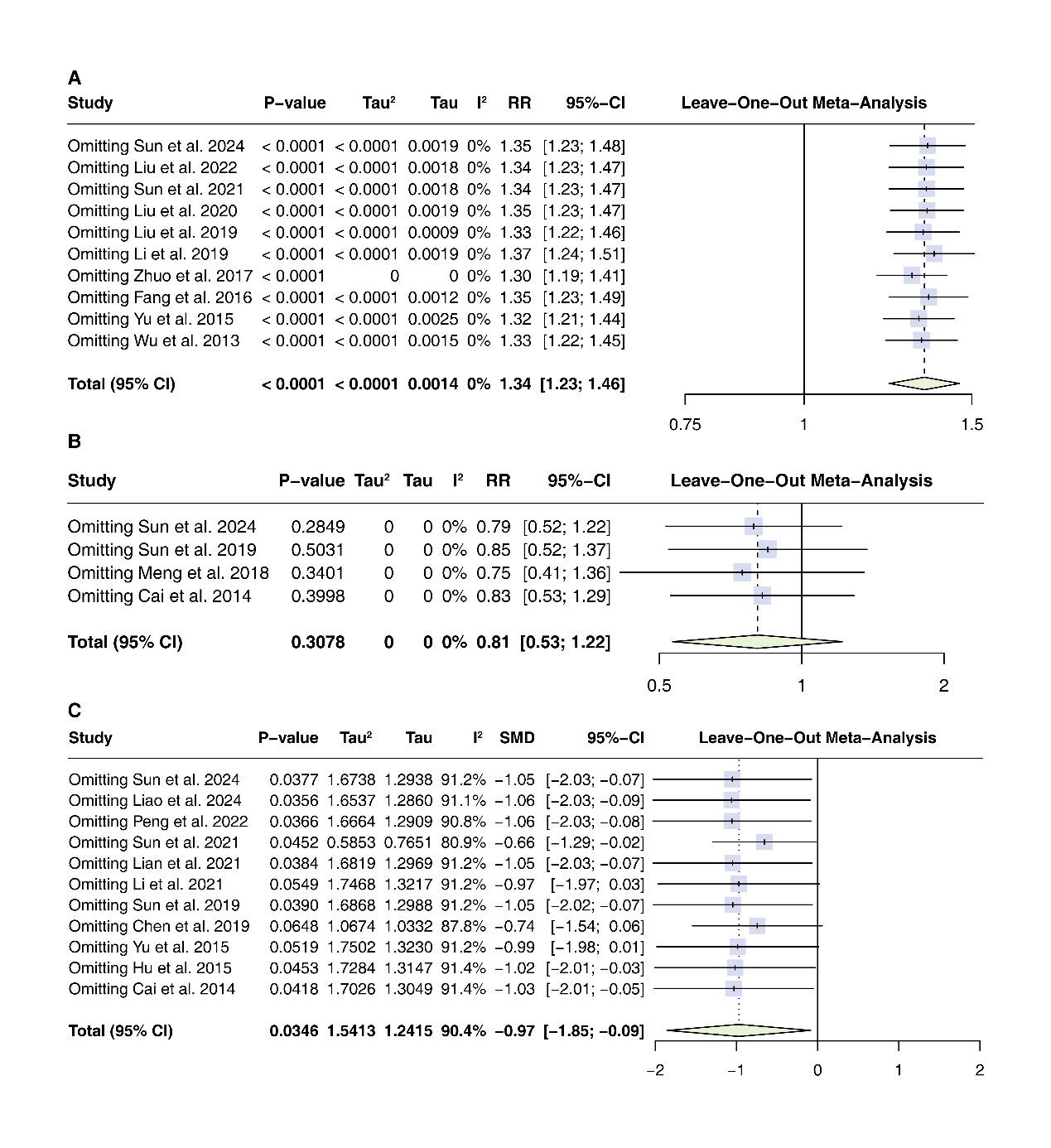


**Figure S2. The results of sensitivity analysis for the clinical effective rate, 28-day mortality and** **APCHE II score.**

Note: A: the clinical effective rate; B: 28-day mortality; C: APCHE II score. CIs, conﬁdence intervals.

**
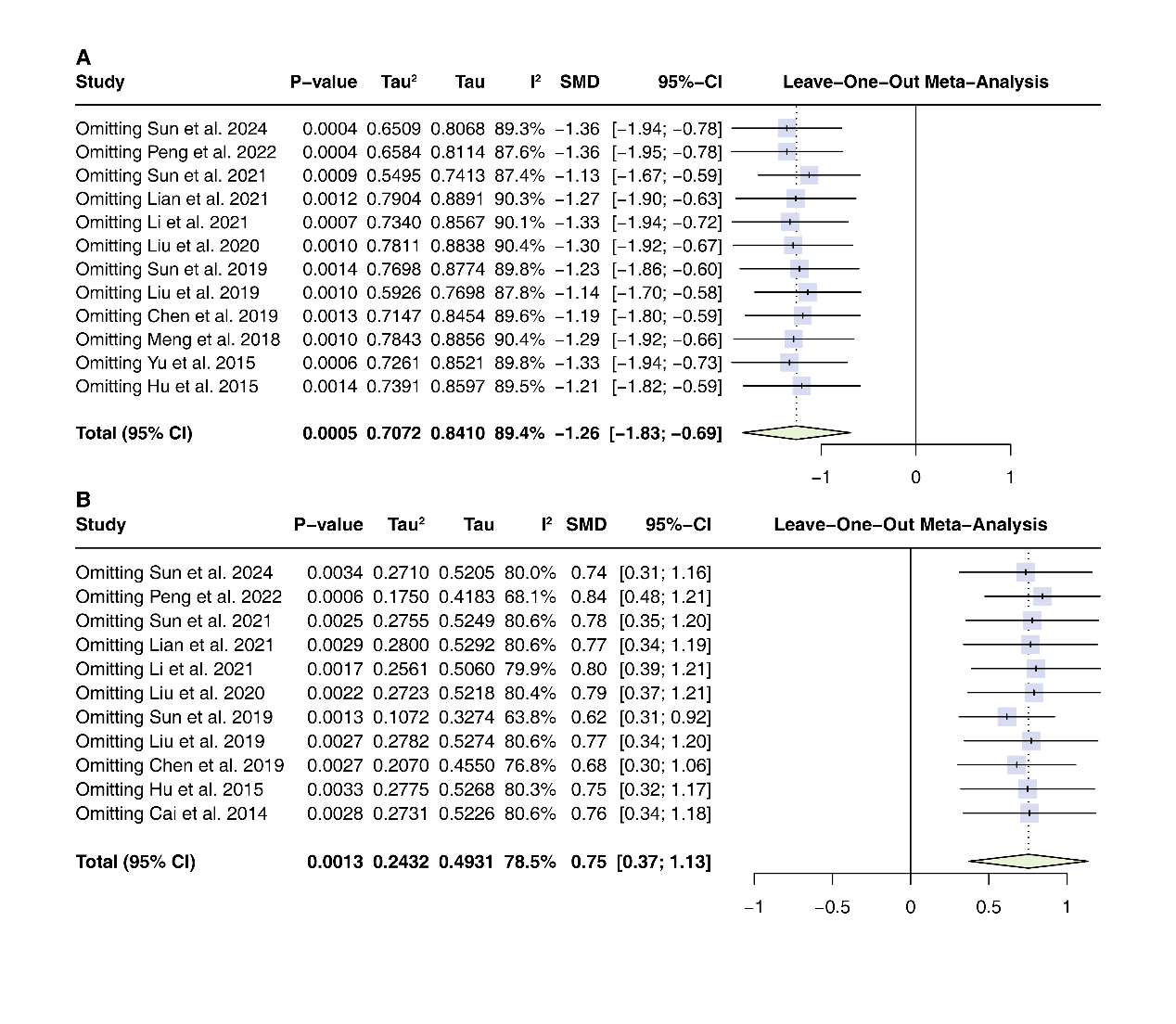
**

**Figure S3. The results of sensitivity analysis for intra-abdominal pressure and bowel sound.**

Note: A: intra-abdominal pressure; B: bowel sound. CIs, conﬁdence intervals.


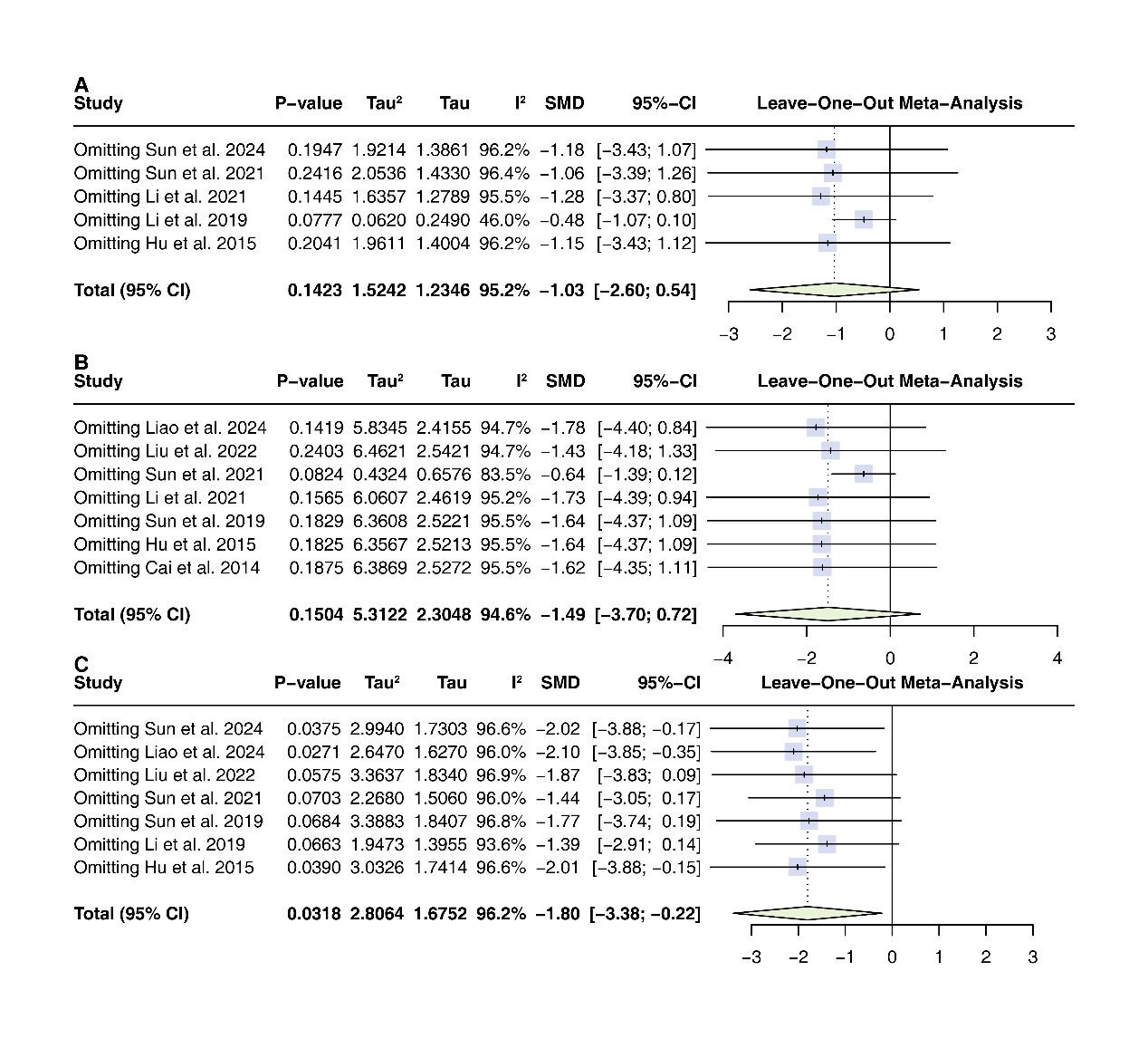


**Figure S4. The results of sensitivity analysis for WBC, CRP and PCT.**

Note: A: WBC; B: CRP; C: PCT. WBC, white blood cell; CRP, C-reactive protein; PCT, procalcitonin; CIs, conﬁdence intervals.


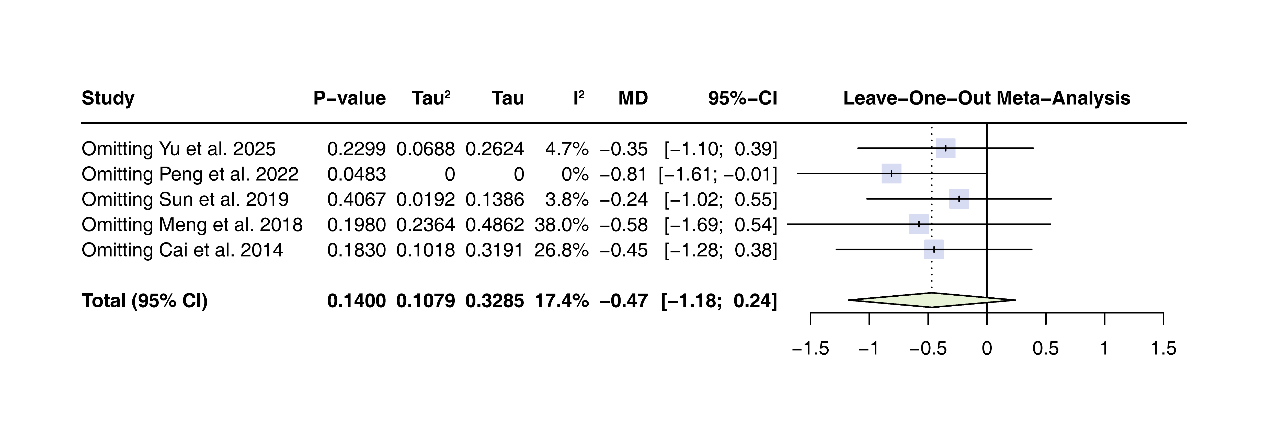


**Figure S5. The results of sensitivity analysis for** **ICU length of stay.**

Note: ICU, intensive care unit; CIs, conﬁdence intervals.


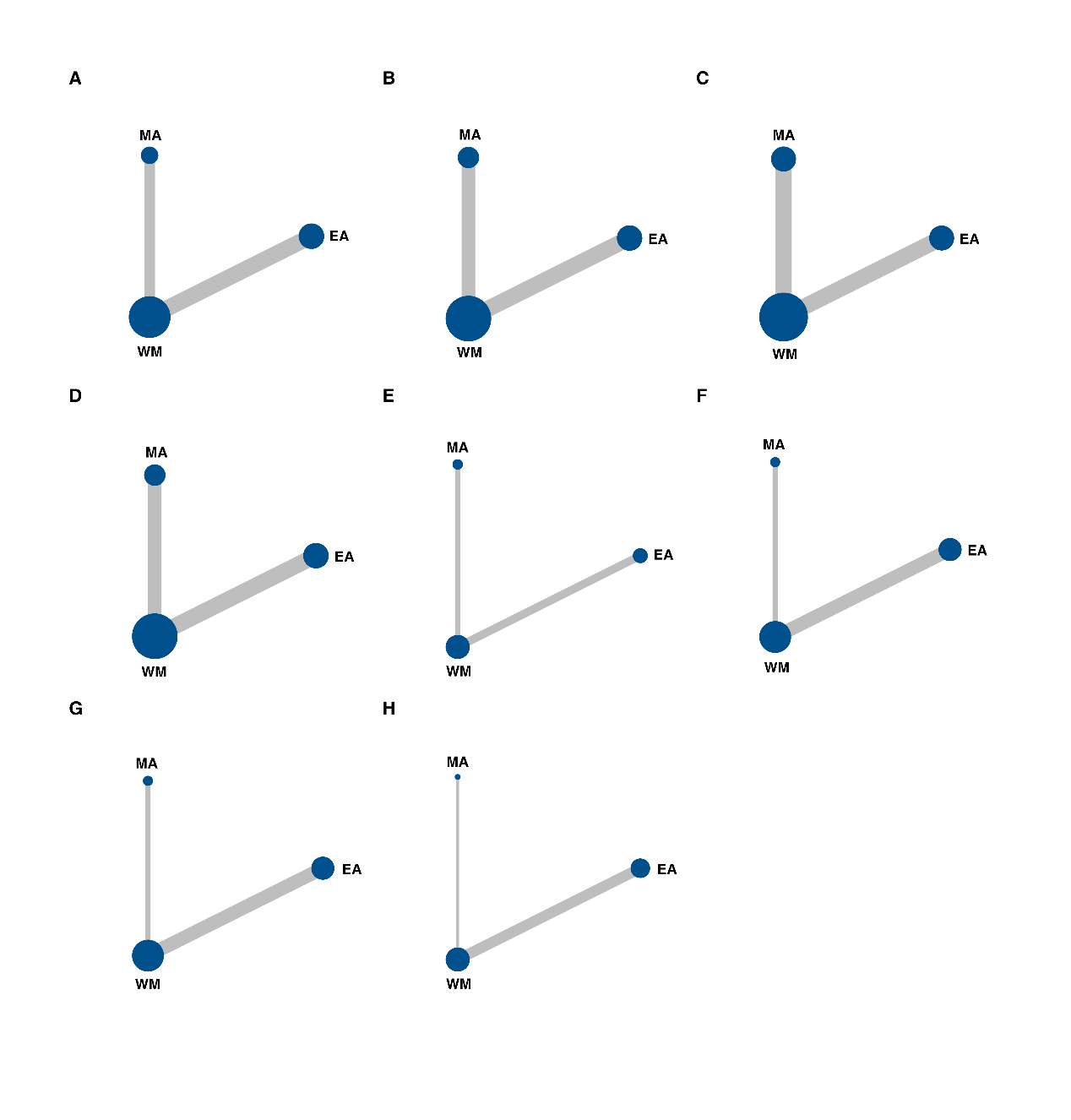


**Figure S6. The Network plot of the different outcomes.**

Note: A: the clinical effective rate; B: APCHE II score; C: intra-abdominal pressure; D: bowel sound; E: WBC; F: CRP; G: PCT. H: ICU length of stay. WBC, white blood cell; CRP, C-reactive protein; PCT, procalcitonin; ICU, intensive care unit.


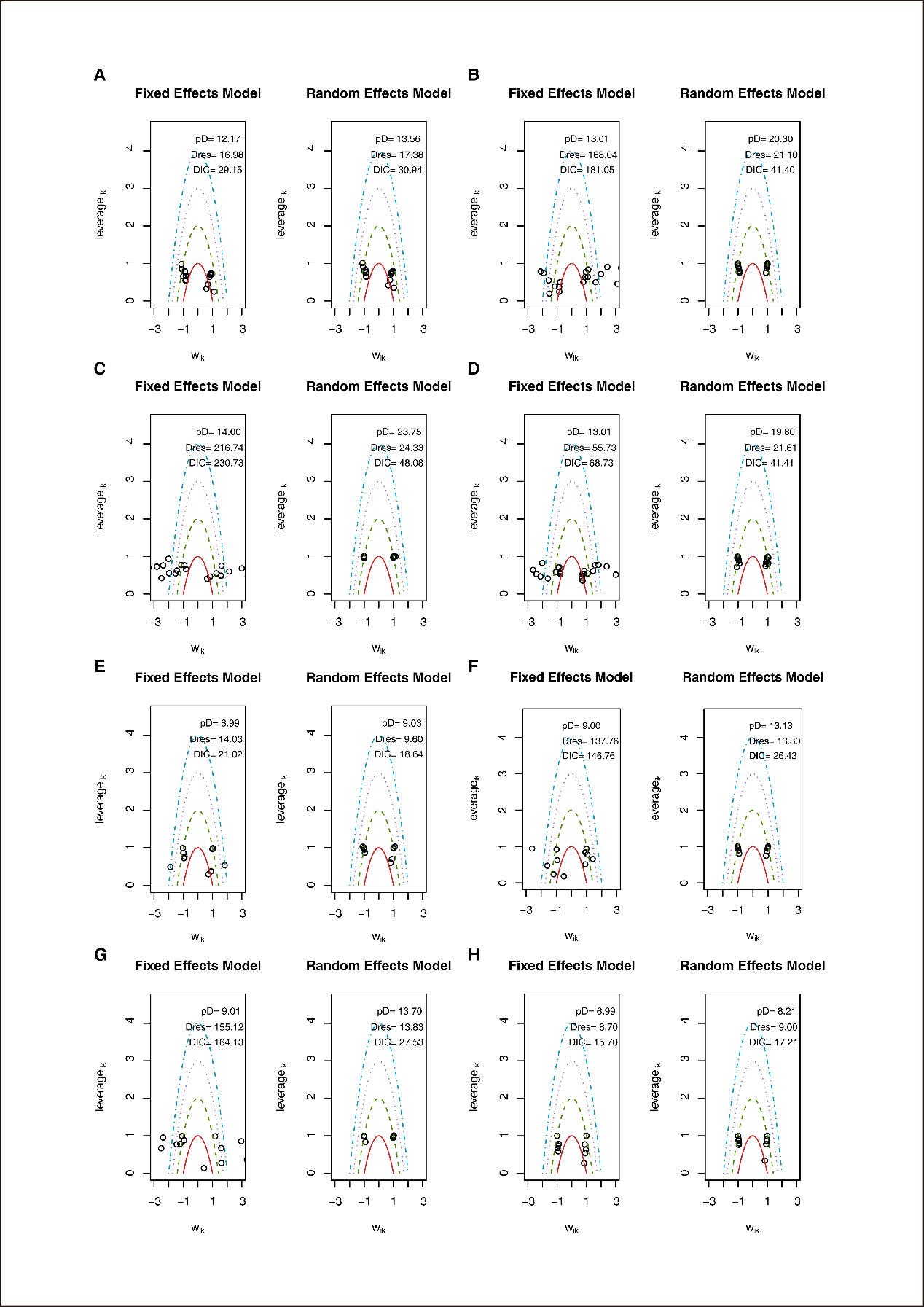


**Figure S7. The Assess model fit of the different outcomes.**

Note: A: the clinical effective rate; B: APCHE II score; C: intra-abdominal pressure; D: bowel sound; E: WBC; F: CRP; G: PCT. H: ICU length of stay. WBC, white blood cell; CRP, C-reactive protein; PCT, procalcitonin; ICU, intensive care unit.

**Table S3. Details of the control group interventions**

| Study | Details of the control group interventions |
| --- | --- |
| Yu et al. 2025 | NA |
| Sun et al. 2024 | Anti-infection therapy, organ function support (including fluid resuscitation, circulatory support, nutritional support, respiratory support, renal replacement therapy, and artificial liver support), as well as immune regulation |
| Liao et al. 2024 | Early fluid resuscitation, vasoactive agents, antimicrobial therapy, mechanical ventilation, and renal replacement therapy; active maintenance of fluid, electrolyte, and acid–base balance; appropriate oxygen therapy; enteral (or parenteral) nutrition; analgesia and sedation; along with other symptomatic supportive treatments |
| Peng et al. 2022 | Anti-infection therapy, protection of organ function, maintenance of stable hemodynamic parameters, and prevention of stress ulcers, among other measures. |
| Liu et al. 2022 | Early fluid resuscitation, anti-infection therapy, glycemic control, maintenance of internal homeostasis, nutritional support, mechanical ventilation, and the use of gastrointestinal prokinetic agents, among other treatments. |
| Sun et al. 2021 | Early fluid resuscitation, vasoactive agents, antimicrobial therapy, mechanical ventilation, and renal replacement therapy; active maintenance of fluid, electrolyte, and acid–base balance; appropriate oxygen therapy; enteral (or parenteral) nutrition; analgesia and sedation; along with other symptomatic supportive treatments |
| Lian et al. 2021 | Early fluid resuscitation, vasoactive agents, antimicrobial therapy, mechanical ventilation, and renal replacement therapy; active maintenance of fluid, electrolyte, and acid–base balance; appropriate oxygen therapy; enteral (or parenteral) nutrition; analgesia and sedation; along with other symptomatic supportive treatments |
| Li et al. 2021 | Early fluid resuscitation, anti-infection therapy, glycemic control, maintenance of internal homeostasis, nutritional support, mechanical ventilation, and the use of gastrointestinal prokinetic agents, among other treatments. |
| Liu et al. 2020 | Early fluid resuscitation, anti-infection therapy, glycemic control, maintenance of internal homeostasis, nutritional support, mechanical ventilation, and the use of gastrointestinal prokinetic agents, among other treatments. |
| Sun et al. 2019 | Early fluid resuscitation, vasoactive agents, antimicrobial therapy, mechanical ventilation, and renal replacement therapy; active maintenance of fluid, electrolyte, and acid–base balance; appropriate oxygen therapy; enteral (or parenteral) nutrition; analgesia and sedation; along with other symptomatic supportive treatments |
| Liu et al. 2019 | Early fluid resuscitation, anti-infection therapy, glycemic control, maintenance of internal homeostasis, nutritional support, mechanical ventilation, and the use of gastrointestinal prokinetic agents, among other treatments. |
| Li et al. 2019 | Early fluid resuscitation, anti-infection therapy, glycemic control, maintenance of internal homeostasis, nutritional support, mechanical ventilation, and the use of gastrointestinal prokinetic agents, among other treatments. |
| Chen et al. 2019 | Early fluid resuscitation, anti-infection therapy, glycemic control, maintenance of internal homeostasis, nutritional support, mechanical ventilation, and the use of gastrointestinal prokinetic agents, among other treatments. |
| Meng et al. 2018 | Early fluid resuscitation, vasoactive agents, antimicrobial therapy, mechanical ventilation, and renal replacement therapy; active maintenance of fluid, electrolyte, and acid–base balance; appropriate oxygen therapy; enteral (or parenteral) nutrition; analgesia and sedation; along with other symptomatic supportive treatments |
| Zhuo et al. 2017 | Early fluid resuscitation, vasoactive agents, antimicrobial therapy, mechanical ventilation, and renal replacement therapy; active maintenance of fluid, electrolyte, and acid–base balance; appropriate oxygen therapy; enteral (or parenteral) nutrition; analgesia and sedation; along with other symptomatic supportive treatments |
| Fang et al. 2016 | Anti-infection therapy, vasoactive agents, fluid management, mechanical ventilation, and other therapeutic measures. |
| Yu et al. 2015 | Early fluid resuscitation, anti-infection therapy, glycemic control, maintenance of internal homeostasis, nutritional support, mechanical ventilation, and the use of gastrointestinal prokinetic agents, among other treatments. |
| Hu et al. 2015 | Anti-infection therapy, vasoactive agents, fluid management, mechanical ventilation, and other therapeutic measures. |
| Cai et al. 2014 | Anti-infection therapy, anti-shock management, respiratory support, and nutritional support, among other treatments. |
| Wu et al. 2013 | Anti-infection therapy, anti-shock management, respiratory support, and nutritional support, among other treatments. |
