# Supplementary material for: Adjunctive acupuncture for sepsis-associated acute gastrointestinal injury: a systematic review, meta-analysis, and exploratory Bayesian network meta-analysis
Source: Front Med (Lausanne). 2026 May 22;13:1806453. doi: 10.3389/fmed.2026.1806453 (PMC13237696; doi:10.3389/fmed.2026.1806453)
Supplement: Supplementary file 2 [file Supplementary_file_1.docx]

**Search Strategy**

Pubmed

| Number | Search terms | Results |
| --- | --- | --- |
| #1 | Sepsis [MeSH Terms] | 153,444 |
| #2 | Septic shock [MeSH Terms] | 26,212 |
| #3 | Systemic inflammatory response syndrome [MeSH Terms] | 162,704 |
| #4 | Sepsis [Title/Abstract] | 141,565 |
| #5 | Septic shock [Title/Abstract] | 31,836 |
| #6 | Systemic inflammatory response syndrome [Title/Abstract] | 6,811 |
| #7 | SIRS [Title/Abstract] | 7,620 |
| #8 | OR/1~7 | 258,088 |
| #9 | Gastrointestinal dysfunction [Title/Abstract] | 1,682 |
| #10 | Acute gastrointestinal injury [Title/Abstract] | 161 |
| #11 | Intestinal failure [Title/Abstract] | 2,949 |
| #12 | OR/9~11 | 4,745 |
| #13 | Acupuncture[mesh] | 33,255 |
| #14 | Pharmacopuncture[Title/Abstract] | 321 |
| #15 | Acupuncture Therapy[mesh] | 32,478 |
| #16 | Acupuncture Treatment OR Acupuncture Treatments OR Treatment, Acupuncture OR Therapy, Acupuncture OR Pharmacoacupuncture Treatment OR Treatment, Pharmacoacupuncture OR Pharmacoacupuncture Therapy OR Therapy, Pharmacoacupuncture [Title/Abstract] | 44,953 |
| #17 | Electroacupuncture[mesh] | 5,760 |
| #18 | Acupuncture Points[mesh] | 8,985 |
| #19 | Acupuncture Point OR Point, Acupuncture OR Points, Acupuncture OR Acupoints OR Acupoint [Title/Abstract] | 17,665 |
| #20 | OR/13~19 | 46,945 |
| #21 | Clinical [Title/Abstract] | 5,186,269 |
| #22 | Trial [Title/Abstract] | 920,971 |
| #23 | 21 AND 22 | 449,527 |
| #24 | clinical trial [Publication Type] | 1,036,932 |
| #25 | randomized controlled trial [Publication Type] | 651,133 |
| #26 | controlled clinical trial [Publication Type] | 741,966 |
| #27 | random*[Title/Abstract] | 1,694,364 |
| #28 | OR/23~27 | 2,349,623 |
| #29 | #8 AND #12 AND #20 AND #28 | 7 |

Web of science

| Number | Search terms | Results |
| --- | --- | --- |
| #1 | TS=(Sepsis OR Septic shock OR Systemic inflammatory response syndrome OR SIRS) | 183,076 |
| #2 | TS=(Gastrointestinal dysfunction OR Acute gastrointestinal injury OR Intestinal failure) | 25,602 |
| #3 | TS=(Acupuncture OR Electroacupuncture OR Pharmacopuncture) | 26,226 |
| #4 | TS=(Acupuncture Therapy OR Acupuncture Treatment OR Acupuncture Treatments OR Treatment, Acupuncture OR Therapy, Acupuncture OR Pharmacoacupuncture Treatment OR Treatment, Pharmacoacupuncture OR Pharmacoacupuncture Therapy OR Therapy, Pharmacoacupuncture) | 16,909 |
| #5 | TS=(Acupuncture Points OR Acupuncture Point OR Point, Acupuncture OR Points, Acupuncture OR Acupoints OR Acupoint) | 8,958 |
| #6 | OR/3~5 | 27,224 |
| #7 | #1 AND #2 AND #6 | 7 |
| #8 | #7 NOT TI=(animal) | 7 |
| #9 | #8 NOT TI=(mice) | 7 |
| #10 | #9 NOT TI=(rat) | 7 |
| #11 | #10 NOT TI=(rabbit) | 7 |

Cochrane Library

| Number | Search terms | Results |
| --- | --- | --- |
| #1 | MESH DESCRIPTOR Sepsis EXPLODE ALL TREES | 6,664 |
| #2 | MESH DESCRIPTOR shock, Septic EXPLODE ALL TREES | 1,485 |
| #3 | MESH DESCRIPTOR Systemic inflammatory response syndrome EXPLODE ALL TREES | 7,204 |
| #4 | (Sepsis OR Septic shock OR Systemic inflammatory response syndrome OR SIRS) :TI,AB,KW | 18,660 |
| #5 | OR/1~4 | 20,759 |
| #6 | MESH DESCRIPTOR Intestinal failure EXPLODE ALL TREES | 21 |
| #7 | (Gastrointestinal dysfunction OR Acute gastrointestinal injury OR Intestinal failure) :TI,AB,KW | 4,339 |
| #8 | #6 OR #7 | 4,339 |
| #9 | MESH DESCRIPTOR Acupuncture EXPLODE ALL TREES | 216 |
| #10 | MESH DESCRIPTOR Acupuncture Therapy EXPLODE ALL TREES | 7,335 |
| #11 | MESH DESCRIPTOR Electroacupuncture EXPLODE ALL TREES | 1,220 |
| #12 | MESH DESCRIPTOR Acupuncture Points EXPLODE ALL TREES | 2,915 |
| #13 | ((Acupuncture OR Acupoint* OR Meridian*)):TI,AB,KW | 25,620 |
| #14 | ((Electroacupuncture OR Electro-acupuncture)):TI,AB,KW | 4,201 |
| #15 | ((Acupunctur* OR Needling OR Acup* point*)):TI,AB,KW | 25,727 |
| #16 | OR/9~15 | 29,658 |
| #17 | #5 AND #8 AND #16 | 21 |

Embase

| Number | Search terms | Results |
| --- | --- | --- |
| #1 | sepsis:ti,ab,kw OR 'septic shock':ti,ab,kw OR 'systemic inflammatory response syndrome':ti,ab,kw OR sirs:ti,ab,kw | 268,931 |
| #2 | 'gastrointestinal dysfunction':ti,ab,kw OR 'acute gastrointestinal injury':ti,ab,kw OR 'intestinal failure':ti,ab,kw | 8,410 |
| #3 | 'acupuncture'/exp OR 'acupuncture points'/exp OR 'points, acupuncture' | 70,439 |
| #4 | 'acupuncture therapy'/exp OR 'acupuncture treatment' OR 'acupuncture treatments' OR 'treatment, acupuncture' OR 'therapy, acupuncture' | 68,728 |
| #5 | 'electroacupuncture'/exp OR 'meridians'/exp | 13,908 |
| #6 | acupuncture:ti,ab,kw | 47,325 |
| #7 | electroacupuncture:ti,ab,kw | 10,508 |
| #8 | acupoint:ti,ab,kw | 6,468 |
| #9 | meridian:ti,ab,kw | 7,362 |
| #10 | deqi:ti,ab,kw | 380 |
| #11 | OR/3~10 | 79,285 |
| #12 | #1 AND #2 AND #11 | 10 |

CNKI

| Number | Search terms | Results |
| --- | --- | --- |
| #1 | SU=sepsis | 32,368 |
| #2 | SU= 'Gastrointestinal dysfunction' OR 'Gastrointestinal disorder' OR 'Gastrointestinal impairment' OR 'Gastrointestinal injury' OR 'Intestinal dysfunction' | 2,186 |
| #3 | SU= Acupuncture | 140,060 |
| #4 | SU= Needle | 3,155 |
| #5 | SU= manul Acupuncture | 736 |
| #6 | SU= Electroacupuncture | 40,273 |
| #7 | OR/3~6 | 40,273 |
| #8 | #1 AND #2 AND #7 | 84 |

WANFANG

| Number | Search terms | Results |
| --- | --- | --- |
| #1 | Topic:(sepsis) | 144,527 |
| #2 | Topic:( 'Gastrointestinal dysfunction' OR 'Gastrointestinal disorder' OR 'Gastrointestinal impairment' OR 'Gastrointestinal injury' OR 'Intestinal dysfunction') | 293,963 |
| #3 | Topic:( 'Acupuncture' OR 'Needle' OR 'manul Acupuncture' OR 'Electroacupuncture') | 322,150 |
| #4 | #1 AND #2 AND #3 | 127 |

VIP

| Number | Search terms | Results |
| --- | --- | --- |
| #1 | M=(sepsis) | 27,242 |
| #2 | R=(sepsis) | 31,118 |
| #3 | #1 OR #2 | 33,382 |
| #4 | M= ('Gastrointestinal dysfunction' OR 'Gastrointestinal disorder' OR 'Gastrointestinal impairment' OR 'Gastrointestinal injury' OR 'Intestinal dysfunction') | 13,005 |
| #5 | R= ('Gastrointestinal dysfunction' OR 'Gastrointestinal disorder' OR 'Gastrointestinal impairment' OR 'Gastrointestinal injury' OR 'Intestinal dysfunction') | 40,927 |
| #6 | #4 OR #5 | 43,536 |
| #7 | M= ('Acupuncture' OR 'Needle' OR 'manul Acupuncture' OR 'Electroacupuncture') | 179,849 |
| #8 | R= ('Acupuncture' OR 'Needle' OR 'manul Acupuncture' OR 'Electroacupuncture') | 194,002 |
| #9 | #7 OR #8 | 233,225 |
| #10 | #3 AND #6 AND #9 | 73 |

SINOMED

| Number | Search terms | Results |
| --- | --- | --- |
| #1 | "sepsis"[Common Field: Intelligent] | 48519 |
| #2 | ('Gastrointestinal dysfunction' OR 'Gastrointestinal disorder' OR 'Gastrointestinal impairment' OR 'Gastrointestinal injury' OR 'Intestinal dysfunction') [Common Field: Intelligent] | 31364 |
| #3 | ('Acupuncture' OR 'Needle' OR 'manul Acupuncture' OR 'Electroacupuncture') [Common Field: Intelligent] | 285768 |
| #4 | #1 AND #2 AND #3 | 95 |

ICMCTR

| Number | Search terms | Results |
| --- | --- | --- |
| 1 | sepsis AND ('Gastrointestinal dysfunction' OR 'Gastrointestinal disorder' OR 'Gastrointestinal impairment' OR 'Gastrointestinal injury' OR 'Intestinal dysfunction') AND ('Acupuncture' OR 'Needle' OR 'manul Acupuncture' OR 'Electroacupuncture') | 3 |

ClinicalTrials.gov

| Number | Search terms | Results |
| --- | --- | --- |
| 1 | sepsis AND ('Gastrointestinal dysfunction' OR 'Gastrointestinal disorder' OR 'Gastrointestinal impairment' OR 'Gastrointestinal injury' OR 'Intestinal dysfunction') AND ('Acupuncture' OR 'Needle' OR 'manul Acupuncture' OR 'Electroacupuncture') | 1 |

ChiCTR

| Number | Search terms | Results |
| --- | --- | --- |
| 1 | sepsis AND ('Gastrointestinal dysfunction' OR 'Gastrointestinal disorder' OR 'Gastrointestinal impairment' OR 'Gastrointestinal injury' OR 'Intestinal dysfunction') AND ('Acupuncture' OR 'Needle' OR 'manul Acupuncture' OR 'Electroacupuncture') | 9 |
